# Differential effects of mutations of POPDC proteins on heteromeric interaction and membrane trafficking

**DOI:** 10.1186/s40478-022-01501-w

**Published:** 2023-01-09

**Authors:** Alexander H. Swan, Roland F. R. Schindler, Marco Savarese, Isabelle Mayer, Susanne Rinné, Felix Bleser, Anne Schänzer, Andreas Hahn, Mario Sabatelli, Francesco Perna, Kathryn Chapman, Mark Pfuhl, Alan C. Spivey, Niels Decher, Bjarne Udd, Giorgio Tasca, Thomas Brand

**Affiliations:** 1 National Heart and Lung Institute (NHLI), Imperial College London, London, UK; 2 Department of Chemistry, Imperial College London, London, UK; 3 Department of Medical Genetics, Medicum, University of Helsinki, Helsinki, Finland; 4 Institute for Physiology and Pathophysiology, Vegetative Physiology, Philipps-University of Marburg, Marburg, Germany; 5 Institute of Neuropathology, Justus Liebig University Giessen, Giessen, Germany; 6 Department of Child Neurology, Justus Liebig University Giessen, Giessen, Germany; 7 Department of Neurology, Universitá Cattolica del Sacro Cuore, Rome, Italy; 8 Dipartimento Di Scienze Cardiovascolari, Fondazione Policlinico Universitario A. Gemelli IRCCS, Rome, Italy; 9 Assay Biology, Domainex Ltd, Cambridge, CB10 1XL UK; 10 School of Cardiovascular Medicine and Sciences and Randall Centre, King’s College London, London, UK; 11 Folkhälsan Research Center, University of Helsinki, Helsinki, Finland; 12 Unità Operativa Complessa di Neurologia, Fondazione Policlinico Universitario A. Gemelli IRCCS, Rome, Italy; 13 Present Address: John Walton Muscular Dystrophy Research Centre, Newcastle University and Newcastle Hospitals NHS Foundation Trusts, Newcastle Upon Tyne, UK; 14 Imperial Centre of Translational and Experimental Medicine, Du Cane Road, London, W120NN UK

**Keywords:** Popeye domain, Limb-girdle muscular dystrophy, Protein–protein interaction, Membrane trafficking, Mutation, α-helix, Cyclic nucleotide binding domain

## Abstract

The Popeye domain containing (POPDC) genes encode sarcolemma-localized cAMP effector proteins. Mutations in blood vessel epicardial substance (*BVES*) also known as *POPDC1* and *POPDC2* have been associated with limb-girdle muscular dystrophy and cardiac arrhythmia. Muscle biopsies of affected patients display impaired membrane trafficking of both POPDC isoforms. Biopsy material of patients carrying mutations in *BVES* were immunostained with POPDC antibodies. The interaction of POPDC proteins was investigated by co-precipitation, proximity ligation, bioluminescence resonance energy transfer and bimolecular fluorescence complementation. Site-directed mutagenesis was utilised to map the domains involved in protein–protein interaction. Patients carrying a novel homozygous variant, *BVES* (c.547G > T, p.V183F) displayed only a skeletal muscle pathology and a mild impairment of membrane trafficking of both POPDC isoforms. In contrast, variants such as *BVES* p.Q153X or *POPDC2* p.W188X were associated with a greater impairment of membrane trafficking. Co-transfection analysis in HEK293 cells revealed that POPDC proteins interact with each other through a helix-helix interface located at the C-terminus of the Popeye domain. Site-directed mutagenesis of an array of ultra-conserved hydrophobic residues demonstrated that some of them are required for membrane trafficking of the POPDC1–POPDC2 complex. Mutations in POPDC proteins that cause an impairment in membrane localization affect POPDC complex formation while mutations which leave protein–protein interaction intact likely affect some other essential function of POPDC proteins.

**Supplementary Information:**

The online version contains supplementary material available at 10.1186/s40478-022-01501-w.

## Introduction

The Popeye domain containing (POPDC) gene family consists of three family members, blood vessel epicardial substance (*BVES*, also known as *POPDC1*), *POPDC2*, and *POPDC3* [2, 35]. POPDC genes encode transmembrane proteins, which are abundantly expressed in the sarcolemma of cardiac and skeletal muscle cells [44]. POPDC proteins consist of a short extracellular amino-terminus, which is subject to N-glycosylation followed by three transmembrane domains [26]. The cytoplasmic part of the protein consists of the Popeye domain and a carboxy-terminus, which is isoform-specific and of variable length [44]. The Popeye domain binds 3′,5′-cyclic adenosine monophosphate (cAMP) with high affinity and specificity [14]. In the heart, POPDC1 and POPDC2 are expressed in cardiac myocytes and both isoforms display high expression levels in the cardiac conduction system (CCS) [2, 8, 14, 48]. Consistent with the CCS expression of both isoforms, a nearly identical stress-induced sinus node bradycardia was observed in *Bves* and *Popdc2* knockout (KO) mice [14]. Enhanced vulnerability of the mutant heart in response to ischemia–reperfusion and impaired skeletal muscle regeneration after injury have also been described for the *Bves* KO mutant [1]. Similarly, cardiac arrhythmia and muscular dystrophy are present in the zebrafish *bves* KO mutant and *popdc2* morphants [25, 40]. POPDC proteins function as a novel class of cAMP effector proteins [44] and interactions with other proteins involved in cAMP signaling such as phosphodiesterase 4 (PDE4) and adenylyl cyclase 9 (AC9) have recently been reported [4, 47].

A sizable number of patients who carry pathogenic variants in POPDC genes have been identified and suffer from heart and/or muscle disease [11, 37, 40, 50, 53]. Patients carrying *BVES* mutations develop a recessive form of limb-girdle muscular dystrophy (LGMDR25), with most patients also suffering from cardiac arrhythmia (sinus bradycardia, sinus tachycardia, or atrioventricular (AV)-block) [5, 10, 11, 15, 19, 40]. Some patients display structural changes in the heart including thickening of the septum and dilated cardiomyopathy [10, 19]. Heart disease with no apparent skeletal muscle involvement has been described in one family [15]. It can be concluded that the pathology caused by *BVES* mutations displays high variability regarding the age of onset, phenotype severity and affected organs. Several reports described a loss of sarcolemmal expression of both POPDC1 and POPDC2 when skeletal muscle biopsies of patients carrying mutations in *BVES* were investigated [11, 19, 40].

In this study, we report a novel recessive mutation in *BVES* (c.547G > T, p.V183F) which has been discovered in two unrelated patients suffering from limb-girdle muscular dystrophy (LGMD) with no cardiac involvement. In contrast to the loss of sarcolemmal expression described for other *BVES* variant cases, expression of POPDC1 and POPDC2 was only weakly diminished in biopsy material of both patients. Currently, it is unclear what determines membrane trafficking of POPDC proteins. We established by co-transfection analysis in HEK293 cells that co-expression of both POPDC1 and POPDC2 was required for proper membrane localization. POPDC proteins undergo heteromeric complex formation as demonstrated by proximity ligation and bioluminescence resonance energy transfer (BRET) analysis. Membrane trafficking is controlled by the formation of an interface between α-helices located at the carboxy-terminus of the Popeye domain of POPDC1 and POPDC2. Modelling identified an array of ultra-conserved hydrophobic residues in both isoforms. Support for their possible involvement in mediating membrane trafficking of POPDC1 and POPDC2 was obtained by site-directed mutagenesis. We propose a model to explain the differential effect of different *BVES* and *POPDC2* variants on membrane trafficking.

## Material and methods

### Study subjects, clinical and molecular examinations

Two unrelated patients affected by a primary muscle disorder were genetically investigated. Genomic DNA was isolated from their blood cells using standard techniques. DNA samples of patients one (PT1) and two (PT2) were sequenced using a targeted gene panel (Myocap) [12] and a clinical exome filtered for 206 myopathy-associated genes respectively. Raw NGS data were analyzed using a standard pipeline. *BVES* variants, described on transcript NM_001199563, were confirmed by PCR and Sanger sequencing (primers available on request) and their segregation analysis was performed on the available family members.

### Animal work

All experiments were performed using age-matched mice. C57BL/6J mice (RRID: IMSR_JAX:000664) were purchased from Harlan UK. The mice were housed in standard cages with a 12-h light/12-h dark cycle at 22–24 °C and ad libitum access to food and water. A *Popdc2* p.W188X knockin (KI) mutation was generated by homologous recombination in embryonic stem cells [37]. *Popdc1*/*Popdc2* double KO animals were generated by crossing *Popdc1* and *Popdc2* KO animals [14]. Both lines were at least ten times backcrossed with C57Bl6J mice and were kept subsequently in a homozygous state. Heart and skeletal muscle tissue of mutant and wild-type mice was obtained after euthanasia.

### Immunostaining

Muscle biopsies from patients and mouse skeletal muscle were processed according to standard procedures [40, 45]. Sections were mounted on Superfrost glass slides (Thermo Fisher Scientific) and subjected to immunohistochemistry using the following primary antibodies: POPDC1 (HPA018176, Sigma-Aldrich), POPDC2 (HPA024255, Sigma-Aldrich), α-sarcoglycan (SGCA, NCL-α-SARC, Leica Biosystems). For the detection of primary antibodies, the following secondary antibodies were employed: Alexa Fluor 488-conjugated donkey anti-rabbit (A21206, Invitrogen) and Alexa Fluor 555-conjugated donkey anti-mouse (A31570, Invitrogen). For counterstaining, 4′, 6-diamidino-2-phenylindole dihydrochloride (DAPI; Calbiochem) was employed. Sections were imaged using a Zeiss LSM 780 AxioObserver inverted confocal laser scanning microscope, with a plan-apochromat 20X/0.8 M27 objective (Zeiss). Three channels were used during image acquisition: 405_ex_/410–495_em_ nm for DAPI, 488_ex_/489–552_em_ nm for Alexa Fluor 488, 543_ex_/548–697_em_ nm for Alexa Fluor 555. 3–15 images were taken for each analysis group.

### Image analysis of skeletal muscle fibers

Images of the immunohistochemically stained skeletal muscle sections were processed using FIJI [39]. The SGCA channel was used to automatically produce outlines of individual muscle fiber cross-sections through the use of a threshold limit. Dilation of the fiber outlines was used to enclose the sarcolemma, with the cytoplasm defined as the inner area of each fiber. The average intensity of Alexa Fluor 488 (POPDC1 or POPDC2) and Alexa Fluor 555 (SGCA) fluorescence within the sarcolemma and cytoplasm of each fiber was then determined. The SGCA-normalized sarcolemmal expression of POPDC1 and POPDC2 was determined by dividing the Alexa Fluor 488 signal within the sarcolemma compartment by that of Alexa Fluor 555. Additionally, the cross-sectional area of each fiber was recorded.

### Cell culture

HEK293 (DSMZ, RRID: CVCL_0045) and COS-7 (DSMZ, RRID: CVCL_0224) were cultured in Dulbecco’s modified Eagle’s medium (Sigma Aldrich) supplemented with 10% (v/v) fetal bovine serum (Merck). HEK293 and COS-7 cells were transiently transfected using Lipofectamine 2000 (Invitrogen) or calcium phosphate (Promega). Cells were incubated for 24–48-h post-transfection before use.

### Cloning procedures

Full length human *POPDC1* and *POPDC2* cDNAs were inserted into the pECFP-N1/pEYFP-N1 plasmid, to append a C-terminus ECFP/EYFP tag. The clinically identified POPDC variants, and the aspartic acid scanning mutations of hydrophobic residues in the αC-helix, were introduced into these constructs using the Q5 site-directed mutagenesis kit (NEB). The oligonucleotide primer sequences used for the site-directed mutagenesis are listed in Additional file 1: Table S1. For NanoBRET analysis, human *POPDC1* and *POPDC2* cDNA sequences were cloned into the pFC14K or pFC32K plasmids (Promega), which contain C-terminus sequences for HaloTag and NanoLuc tags, respectively, using the SgfI and EcoICRI restriction sites. For bimolecular fluorescence complementation (BiFC), split Venus VN155 and VC155 tags (kindly provided by Carmen Dessauer, University of Texas, Houston) were ligated into pECFP-N1 or pEYFP-N1 plasmids containing full-length wild-type and mutant POPDC cDNA sequences using NotI and BamHI restriction sites.

### Quantitative BRET

Type-1 quantitative bioluminescence resonance energy transfer (qBRET) experiments utilized the NanoBRET platform (Promega) and followed the general type-1 qBRET protocol previously reported [13]. The assay was performed in HEK293 cells transiently expressing POPDC1 and POPDC2 constructs which possessed a C-terminal NanoLuc luciferase or HaloTag domains. 100 nM of HaloTag-618 dye (Promega) was added to the cells 24-h before BRET measurement, with an equal number of cells receiving only dimethylsulfoxide (DMSO) to enable the determination of background BRET, which was subtracted from final BRET values. Cells were placed in white 96-well tissue culture plates, immersed in OptiMEM I reduced serum media supplemented with 4% (v/v) fetal bovine serum, 24-h before BRET measurement. BRET was measured using a Lumistar Optima luminometer (BMG Labtech) 5-min after the addition of furimazine NanoLuc substrate (Promega). A range of expression ratios of the NanoLuc and HaloTag containing constructs within the cells was achieved by varying the proportion of each plasmid during transfection, while keeping the total amount constant. Actual expression levels of the NanoLuc- and HaloTag-fused constructs were determined by measuring the total luminescence and HaloTag-618 fluorescence from the cells, respectively. The total expression levels of POPDC isoforms within the cells was determined by summing the normalized NanoLuc luminescence and HaloTag-618 fluorescence. The BRET curves produced were compared to ideal curves for monomers, dimers and other stoichiometries to determine the likely POPDC complex stoichiometry according to a method previously reported [13].

### Co-expression of POPDC isoforms in HEK293 cells

HEK293 cells transiently expressing POPDC1 and POPDC2 constructs tagged at the C-terminal with either enhanced cyan fluorescent protein (ECFP) or enhanced yellow fluorescent protein (EYFP) were incubated with 0.5% (v/v) CellBrite Red solution (Biotium) containing 1,1′-dioctadecyl-3,3,3′,3′-tetramethylindo-dicarbocyanine (DiD) for 12-min at 37 °C to stain the plasma membrane. Cells were washed with PBS before fixation with PFA and stained with Hoechst-33342. Cells were imaged using a LSM 780 AxioObserver inverted confocal laser scanning microscope (Zeiss), with a plan-apochromat 63X/1.40 oil objective (Zeiss). Four channels: 405_ex_/410–452_em_ nm, 458_ex_/463–516_em_ nm, 514_ex_/519–621_em_ nm and 633_ex_/636–735_em_ nm, were used to image the Hoechst-33342, POPDC1-ECFP, POPDC2-EYFP and DiD, respectively. Cells were imaged in poly-L-lysine coated 8-well microscope slides with a D263 M Schott glass, No. 1.5H, 170 μm ± 5 μm glass coverslip base (Ibidi) immersed in PBS.

### Image analysis of transfected cells

Images were analyzed using FIJI. The plasma membrane of the HEK293 cells was manually outlined using the DiD channel as a guide, followed by dilation to encompass the entire plasma membrane. The area within the inner edge of the plasma membrane boundary up to the nucleus, as highlighted by Hoechst-33342, was defined as the cytoplasm. Background fluorescence was subtracted from all images before analysis. The intensity of ECFP and EYFP fluorescence intensity with each compartment was then determined to find the concentration of POPDC1 and POPDC2, respectively.

### Proximity ligation assay

A Duolink® proximity ligation assay (PLA; Sigma-Aldrich) was employed using a goat-polyclonal anti-POPDC1 antibody (sc-49889, Santa Cruz Biotechnology) and a polyclonal rabbit anti-POPDC2 antibody (HPA024255, Sigma-Aldrich), on heart sections of wild-type and *Bves/Popdc2* double KO mutants according to the standard protocol. Staining with wheat germ agglutinin and DAPI was used to visualize the sarcolemmal and nuclear compartments, respectively.

### Western blot and co-immunoprecipitation analysis

Cells expressing POPDC1-CFP and/or POPDC2-FLAG, or POPDC1-CFP and/or POPDC3-MYC were lysed 24-h post-transfection using 4 M urea and 10% (w/v) SDS without a reducing agent. Cell lysates were sonicated and centrifuged for 30-min at > 16,000 g. The cleared lysate was incubated at 37 °C for 30-min and subjected to Western blot analysis using an anti-GFP antibody (Abcam) to detect POPDC1-CFP containing complexes. Ventricles of wild-type and *Popdc2* KO mutant mice were excised, snap-frozen in liquid nitrogen and pulverized with a pre-cooled pestle and mortar. The tissue was lysed using a 1% (v/v) Triton X-100 based lysis buffer followed by sonification. The lysates were centrifuged for 30-min at > 16,000 g. Equal protein concentrations were used across all samples. A rabbit anti-POPDC2 antibody (HPA024255, Sigma-Aldrich) was incubated with the cleared lysate overnight. Antibodies were captured using Protein A agarose, which were centrifuged, washed then resuspended in NuPAGE LDS Sample Buffer (Invitrogen) and incubated at 96 °C for 5-min. After removal of the remaining agarose by centrifugation the sample was supplemented with NuPAGE Sample Reducing Agent (Invitrogen) and analyzed by Western blotting. POPDC1 was detected using a polyclonal goat anti-POPDC1 antibody (sc-49889, Santa Cruz Biotechnology). COS-7 cells transiently expressing POPDC constructs with the appropriate epitope tag were lysed using a 1% (v/v) Triton X-100 based lysis buffer supplemented with cOmplete protease inhibitor cocktail (Roche). Lysates were subjected to Co-IP using the ProFoundTM c-Myc Tag IP/co-IP Kit (Thermo Scientific) or the Pierce HA Tag IP/Co-IP Kit (Thermo Scientific), following the manufacturer’s protocols. The antibody conjugated agarose beads were washed, resuspended in sample buffer and analyzed by Western blotting.

### Measurement of TREK-1 current

*Xenopus laevis* were maintained and oocytes isolated under standard conditions according to established protocols. Capped cRNA transcripts were synthesized in vitro using the mMessage mMachine T7 transcription kit (Ambion). The cRNAs were purified and photometrically quantified. cRNA coding for human TREK-1c alone or together with mouse Popdc1 or mouse Popdc2 were injected into *Xenopus laevis* oocytes. Oocytes were incubated at 19 °C for 48-h in ND96 solution containing 96 mM NaCl, 2 mM KCl, 1 mM MgCl_2_, 1.8 mM CaCl_2_, and 5 mM HEPES (pH 7.5) supplemented with 50 mg/l gentamicin and 275 mg/l sodium pyruvate. For experiments with elevated cAMP levels, 25 mM theophylline was supplemented to the storage solution, directly following the cRNA injection. Two-microelectrode voltage-clamp measurements were performed with a Turbo Tec-10 C amplifier (npi, Tamm). The oocytes were placed in a small-volume perfusion chamber and superfused with ND96 solution. Micropipettes were made from borosilicate glass capillaries GB 150TF-8P (Science Products) and pulled with a DMZ-Universal Puller (Zeitz). The resistance of the recording pipettes was 0.5–1.5 MΩ when pipettes were filled with 3 M KCl solution. TREK-current was measured using a voltage step protocol from a holding potential of − 80 mV. A first test pulse to 0 mV of 1 s duration was followed by a repolarizing step to − 80 mV for 1 s, directly followed by another 1 s test pulse to + 40 mV. The sweep time interval was 10 s. Current amplitudes were analyzed at + 40 mV. Since current amplitudes varied from one batch of oocytes to the next, currents were normalized to TREK-1c WT current amplitudes of the respective batch and recording day.

### Bimolecular fluorescence complementation (BiFC)

POPDC1 and POPDC2 were tagged at the C-terminal with split Venus domains VC155 or VN155, respectively, and expressed in HEK293 cells. Co-transfection of POPDC1-VN155 and POPDC2-VN155 was used as a negative control. All cells were also transfected with pmRFP-N1 as an internal control for transfection efficiency. Cells were fixed using PFA and stained with Hoechst-33342. The cells were imaged using an Axio Observer inverted confocal laser scanning microscope (Zeiss) using a 10X objective (Zeiss). Three channels were used during image acquisition: 405_ex_/410–503_em_ nm for Hoechst-33342, 514_ex_/516–587_em_ nm for reformed Venus and 543_ex_/582–754_em_ nm for mRFP. Images were analyzed using FIJI. All cells expressing above-background levels of mRFP fluorescence were selected for analysis from each image, thus excluding non-transfected cells. The median Venus fluorescence from these cells was determined, disregarding the nuclei as defined by Hoechst-33342 staining. The Venus signal from each image was normalized to mRFP fluorescence and the average from each set of images found.

### Sequence alignments and structural models of POPDC proteins

**Fig. 1:**
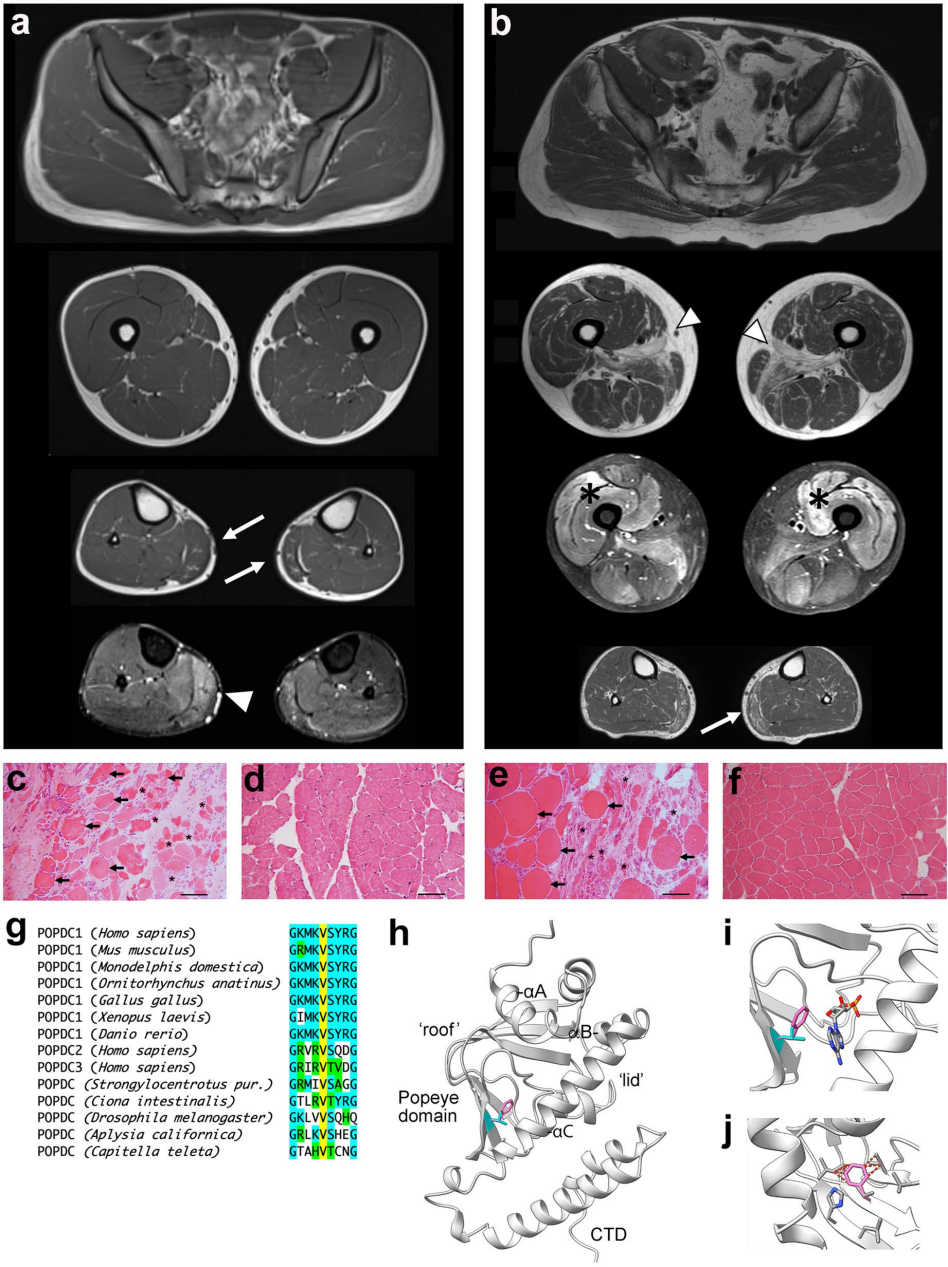
Two non-related patients carrying a *BVES p.V183F* variant and suffering from LGMDR25. **a**, **b** Axial muscle MRI images of **a** patient 1 (PT1) and **b** patient 2 (PT2). PT1 was scanned at age 17 displaying fatty replacement and hypotrophy of the *gastrocnemius medialis* (arrows), with hyperintensity on T2-STIR images (arrowheads). PT2 scanned at age 50 displaying, in addition to changes in the *gastrocnemius medialis* (arrows), also advanced fatty replacement of *adductor longus* greater than in *adductor magnus* (arrowheads) and diffuse T2-STIR hyperintense lesions in the thigh, more evident in the anterior compartment (asterisks). **c**–**f** HE stained transverse sections of muscle biopsies taken from **c** PT1, **e** PT2 and respective matched controls **d** CT1 and **f** CT2. Note the fiber size heterogeneity in both patients. Moderately hypertrophied (arrows) and hypo/atrophied muscle fibers (asterisks) are seen. In PT1, a prominent increase in connective tissue is present. **g** Sequence alignment of part of the Popeye domains of vertebrate and invertebrate POPDC proteins. Color code: V183 (yellow), conserved (turquoise) and similar (green) residues. **h** Model of the Popeye domain of POPDC1 with the position of V183 (cyan) and the mutant V183F (pink) residues highlighted. The position of the phenylalanine side chain was determined using the Dynameomics rotamer library [41]. **i** The position of the V183F mutation relative to the predicted cAMP binding site as determined using the 3DLigandSite server [54]. **j** A model showing the possible steric clashes between the side chain of V183F (pink) with other residues of the β-folds of the Popeye domain as predicted by the Dynameomics rotamer library [41]

For the sequence alignment shown in Fig. 1g, different vertebrate POPDC1 and invertebrate POPDC homologues were identified by BLAST. The sequences used for the alignment have the following accession numbers at the NCBI protein database (https://www.ncbi.nlm.nih.gov/protein): *Homo sapiens* (AAH40502.2), *Mus musculus* (NP_077247.1), *Monodelphis domestica* (XP_016286698), *Ornithoryhnchus anatinus* (XP_028903352.1), *Gallus gallus* (NP_001001299), *Xenopus laevis* (AF527799_1), *Danio rerio* (NP_001244093.1), *Strongylocentrotus purpuratus* (XP_003723894.2), *Ciona intestinalis* (XP_002127439.1), *Drosophila melanogaster* (NP_608426.1), *Aplysia californica* (XP_012939248.2), *Capitella teleta* (ELT88986). AlphaFold Protein Structure Database [49, 51] models of human POPDC1 and POPDC2 were used for modelling purposes and were analyzed using Chimera X [33]. Possible steric clashes caused by the V183F mutation were predicted using the Dynameomics rotamer library within ChimeraX [41]. Overlays of CAP and Popeye domain protein structures were created using the Matchmaker tool in ChimeraX. Predictions of cAMP binding to the Popeye domain were made using the Phyre2 and 3DLigandSite servers [24, 54].

### Statistics

The absolute, or SGCA-normalized, expression levels of POPDC1 and POPDC2 within the sarcolemma and cytoplasm of individual muscle fibers were normalized to the median values from matched control fibers, which were set to equal 1. The normalized median and associated 95% confidence interval (CI) limits of POPDC1 and POPDC2 expression levels in the patient or KI mutant fibers were determined, then compared using a Mann–Whitney test.

The median difference and 95% CI limit in the ratio of POPDC1-ECFP and POPDC2-EYFP constructs at the plasma membrane versus cytoplasm in HEK293 cells was determined and normalized to the median value for cells expressing both wild-type POPDC1 and POPDC2 constructs. Absolute changes in POPDC1 and POPDC2 expression in each compartment were also recorded. The expression levels between single and double wild-type expression groups were analyzed using a Mann–Whitney test, while the effect of the various POPDC mutations, compared to wild-type, were assessed using a Kruskal–Wallis test followed by Dunn’s test.

During the BiFC assay the BiFC signal was defined as the median Venus signal normalized to median mRFP emanating from all transfected cells within each image. The average BiFC signal between the different expression groups were further normalized to the wild-type POPDC pair, set to 1, and compared using a One Way ANOVA followed by Dunnett’s test.

## Results

### A novel POPDC1 p.V183F variant leads to mild loss of POPDC1 and POPDC2 at the sarcolemma

We report here a novel variant in *BVES* (c.547G > T, p.V183F), which has been identified in two unrelated patients. The variant was present in homozygosity in both patients, while the unaffected family members were heterozygous or wild-type. The variant is not expected to cause a mis-splicing (SpliceAI max score 0.02) and is classified in VarSome (https://varsome.com/) as a variant of unknown significance (VUS). However, its identification in two unrelated patients with a similar phenotype suggests that there is sufficient evidence for the variant to be classified as VUS/likely pathogenic.

Patient 1 (PT1) was first investigated at age 17 for asymptomatic hyperCKemia, with values of 3000–3500 UI/L. Muscle MRI of the lower limbs showed bilateral hypotrophy and early fatty changes of the *gastrocnemius medialis*, which was also hyperintense on T2-STIR sequences (Fig. 1a). Muscle biopsy obtained from the same muscle displayed increased fiber size variability and dystrophic changes (Fig. 1c, d) with normal immunostaining for conventional sarcolemmal proteins (data not shown). Regarding the cardiological features, the patient neither complained of suspicious symptoms (fatigue, dyspnea, dizziness, palpitations) nor showed any structural or functional abnormality at baseline and follow-up visits. No arrhythmias were detected at baseline ECG and 24-h Holter monitoring. The echocardiogram and the cardiac MRI showed a structurally normal heart. After almost four years of follow-up, he only reported a single vasovagal syncope triggered by emotional stress, while his Holter ECG showed a para-physiological sinus bradycardia with normal chronotropic competence during the day; the other clinical and instrumental cardiac features remained unchanged. The patient did not agree to undergo invasive tests such as electrophysiological study and loop recorder implantation, which were proposed.

Patient 2 (PT2) had a clinical onset at age 47 with myalgias and burning pain in the lower limbs. After one year, he underwent renal transplantation for chronic kidney failure, likely due to hypertensive nephropathy. One month after transplantation, he developed proximal lower limb weakness, which rapidly progressed in the following years. His serum creatine kinase (CK) level was above 6000 UI/L, with subsequent fluctuations between 2500 and 9000 UI/L, and based upon a suspicion of an immune-mediated myopathy, he was treated with steroids, together with one infusion of intravenous immunoglobulins and chronic cyclosporine treatment, without benefit. Muscle imaging showed fatty replacement of *gluteus minimus*, *adductor longus*, and *magnus*, the left *semimembranosus* and *gastrocnemius medialis* bilaterally, together with relatively widespread abnormalities on T2-STIR images in the thigh muscles, especially in the anterior compartment, and in the *gastrocnemii* (Fig. 1b). An assay for myositis-specific antibodies turned out to be negative. At age 51, he could climb stairs using a handrail but could not raise from a chair without the use of arms. On physical examination, there was weakness of hip and knee flexion on the left side (Medical Research Council grade 4) and of knee extension bilaterally (grade 3). Electromyography was myopathic and nerve conduction studies were normal. Muscle biopsy from the right *vastus lateralis* showed, alongside myopathic changes (Fig. 1e, f), increased endomysial and perimysial fibrosis, and several necrotic fibers with myophagias; hypotrophic round fibers often concentrated in some fascicles; several nuclear clumps, and almost type II fiber uniformity on ATPase stainings were present. HLA class I staining was positive only in necrotic and regenerating fibers, and a mild reduction of sarcolemmal staining for caveolin-3 could be appreciated. Regarding the cardiological features, the patient did not complain of palpitations and did not have any syncope. His ECG was within normal range, only showing mild left axis deviation, and he did not show any arrhythmias on 24-h ECG monitoring; his heart rate was normal throughout the recording. The echocardiogram showed mild, non-pathological interventricular septum hypertrophy (13 mm), which could be explained by hypertension, normal biventricular function, and no functional or structural abnormalities. The patient could not complete cardiac MRI because of claustrophobia. He agreed to undergo electrophysiological study and loop recorder implantation, which however has not yet been performed.

The POPDC1 p.V183F mutation affects a residue that is strongly conserved (Phylo IP100 score = 7.844) and present in all three vertebrate POPDC isoforms and in invertebrate POPDC proteins (Fig. 1g). In the model of the Popeye domain of POPDC1, V183 is located in one of the β-strands (β4) and part of the jelly roll fold forming the roof of the cAMP binding Popeye domain (Fig. 1h). V183 faces into the core of the Popeye domain in close proximity to the predicted cAMP binding pocket. Direct contact between cAMP and V183 is not predicted, although, while the substitution preserves the hydrophobic character at this position, it is unclear if there is any impact on cAMP binding due to steric effects (Fig. 1i). The increased steric demand of phenylalanine may have a structural impact through clashes with other side chains in the β-folds (Fig. 1j). However, modelling the V183F mutation using Missense3D [20] did not predict any major structural aberrations to the Popeye domain.

**Fig. 2:**
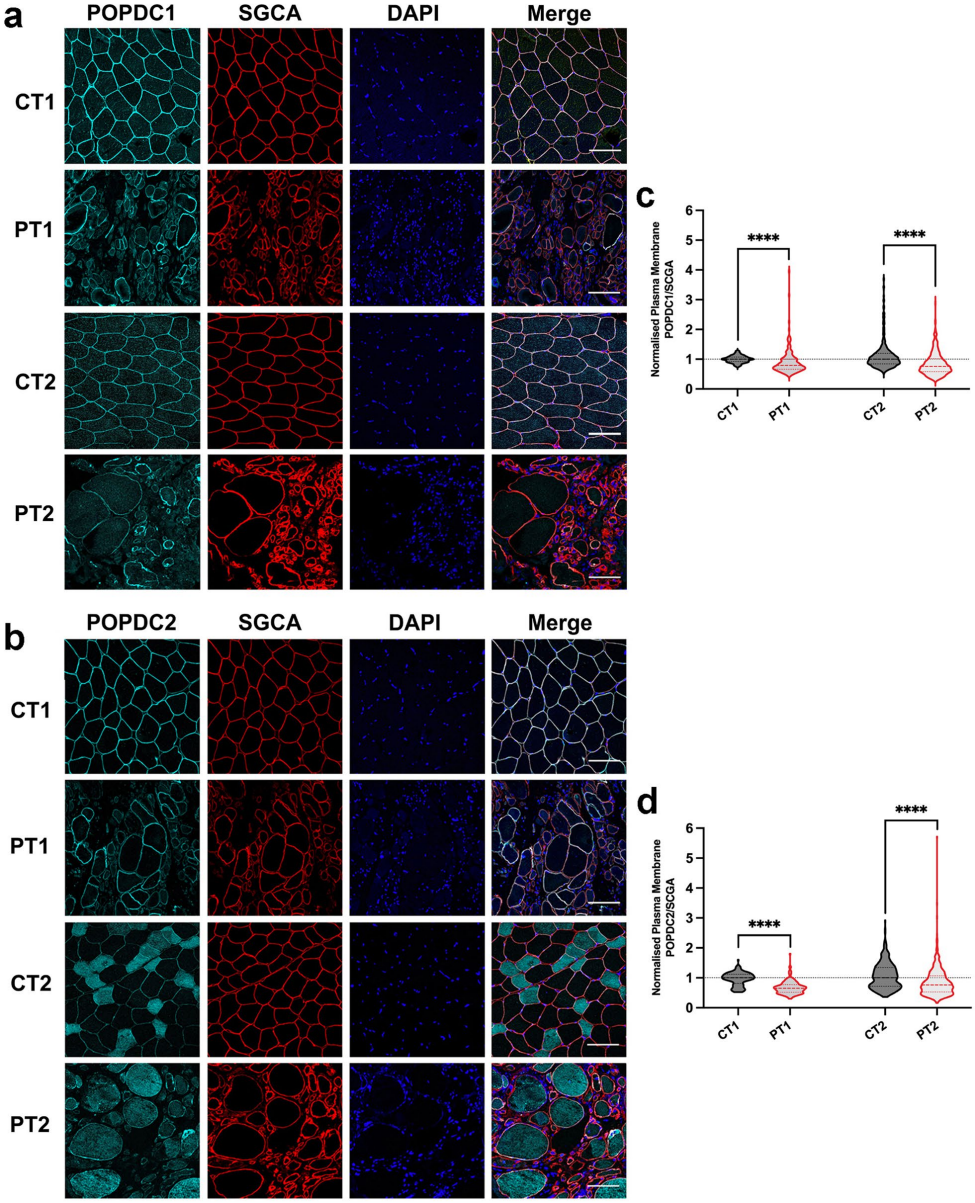
Membrane localization of POPDC isoforms in muscle fibers expressing the POPDC1 p.V183F variant. **a** and **b** Transverse sections of skeletal muscle biopsies from PT1 and PT2 harboring the *POPDC1* p.V183F variant and respective matched controls (CT1 and CT2) were stained for **a** POPDC1 or **b** POPDC2 along with SGCA serving as a sarcolemma marker. Scale bar: 100 μm. **c** and **d** The expression levels of **c** POPDC1 and **d** POPDC2 at the sarcolemma were normalized to SGCA and quantified in individual fibers. The number of sections (sec), images (img) and fibers (fib) analyzed per group are as follows: CT1: POPDC1—2 sec, 5 img, 161 fib; POPDC2—2 sec, 5 img, 138 fib. PT1: POPDC1—1 sec, 4 img, 167 fib; POPDC2—1 sec, 4 img, 167 fibers. CT2: POPDC1—1 sec, 13 img, 681 fib; POPDC2—2 sec, 8 img, 339 fib. PT2: POPDC1—3 sec, 20 img, 835 fib; POPDC2—3 sec, 14 img, 453 fib. The median POPDC/SGCA-level in each control biopsy was set to 1. Dashed lines indicate the normalized median and interquartile range. Data were analyzed using Mann–Whitney test; ****p < 0.0001

Skeletal muscle biopsy material from both patients and from age- and sex-matched controls (CT1 and CT2) were sectioned and stained for either POPDC1 or POPDC2. Sections were also stained for SGCA to mark the sarcolemma of the fibers and served as a control for changes in the expression of POPDC isoforms (Fig. 2a, b), as previously reported [11, 40]. The expression level of POPDC protein and SGCA was measured, with the median level of each control sample set to one, and the differences between the patients and controls analyzed. An approximate 20% reduction (*p* < 0.0001) in the median SGCA-normalized POPDC1 staining intensity in the sarcolemma of fibers of PT1 was observed (0.790, 95% CI 0.744, 0.855; n = 167) compared to CT1 (1.000, 95% CI 0.982, 1.012; n = 161). A similar reduction of around 25% (*p* < 0.0001) was seen in PT2 (0.755, 95% CI 0.731, 0.771; n = 835) compared to CT2 (1.000, 95% CI 0.972, 1.024; n = 681) (Fig. 2c, d). Meanwhile, the SGCA-normalized level of POPDC2 in the sarcolemma was reduced by 35% (*p* < 0.0001) in PT1 (0.650, 95% CI 0.619, 0.681; n = 167) compared to CT1 (1.000, 95% CI 0.951, 1.033; n = 138), with a slightly milder reduction of 24% (*p* < 0.0001) between PT2 (0.763, 95% CI 0.715, 0.810; n = 453) and CT2 (1.000, 95% CI 0.938, 1.077; n = 339) (Fig. 2c, d). Mild reductions in the non-normalized expression levels of both isoforms at the sarcolemma were found (except for POPDC2 in PT2), while an increase in the cytoplasmic concentrations of POPDC2 was also observed in both PT1 and PT2 (Additional file 1: Fig. S1a, b, d, e). These changes led to mild reductions in the enrichment of POPDC1 and POPDC2 at the sarcolemma membrane compared to the cytoplasm, which is representative of how effectively the POPDC proteins are localized at the sarcolemma (Additional file 1: Fig. S1c, f). The changes in POPDC1 and POPDC2 expression were highly variable between individual fibers, with many fibers from the patients resembling control fibers with respect to POPDC protein expression while others showed major differences. A subpopulation of fibers from both PT2 and CT2 displayed a large increase in cytoplasmic levels of POPDC2, as can be seen in Fig. 2b. While the cause of this effect is unknown, it may reflect the increased age of PT2 and the matched control compared to PT1. All fibers were included in the analysis. Irregular and variable fiber sizes and morphologies were seen in both patients (Fig. 2a, b). The median fiber cross-sectional areas were lower in both patients, with an 80% drop (*p* < 0.0001) between CT1 (2968 μm^2^, 95% CI 2843, 3101; n = 296) and PT1 (628 μm^2^, 95% CI 586, 680; n = 327) and a 63% reduction (*p* < 0.0001) between CT2 (3269 μm^2^, 95% CI 3190, 3380; n = 1020) and PT2 (1215 μm^2^, 95% CI 1151, 1322; n = 1288) (Additional file 1: Fig. S1g).

### A POPDC1 p.Q153X mutation leads to a severe loss of POPDC1 and POPDC2 at the sarcolemma

**Fig. 3:**
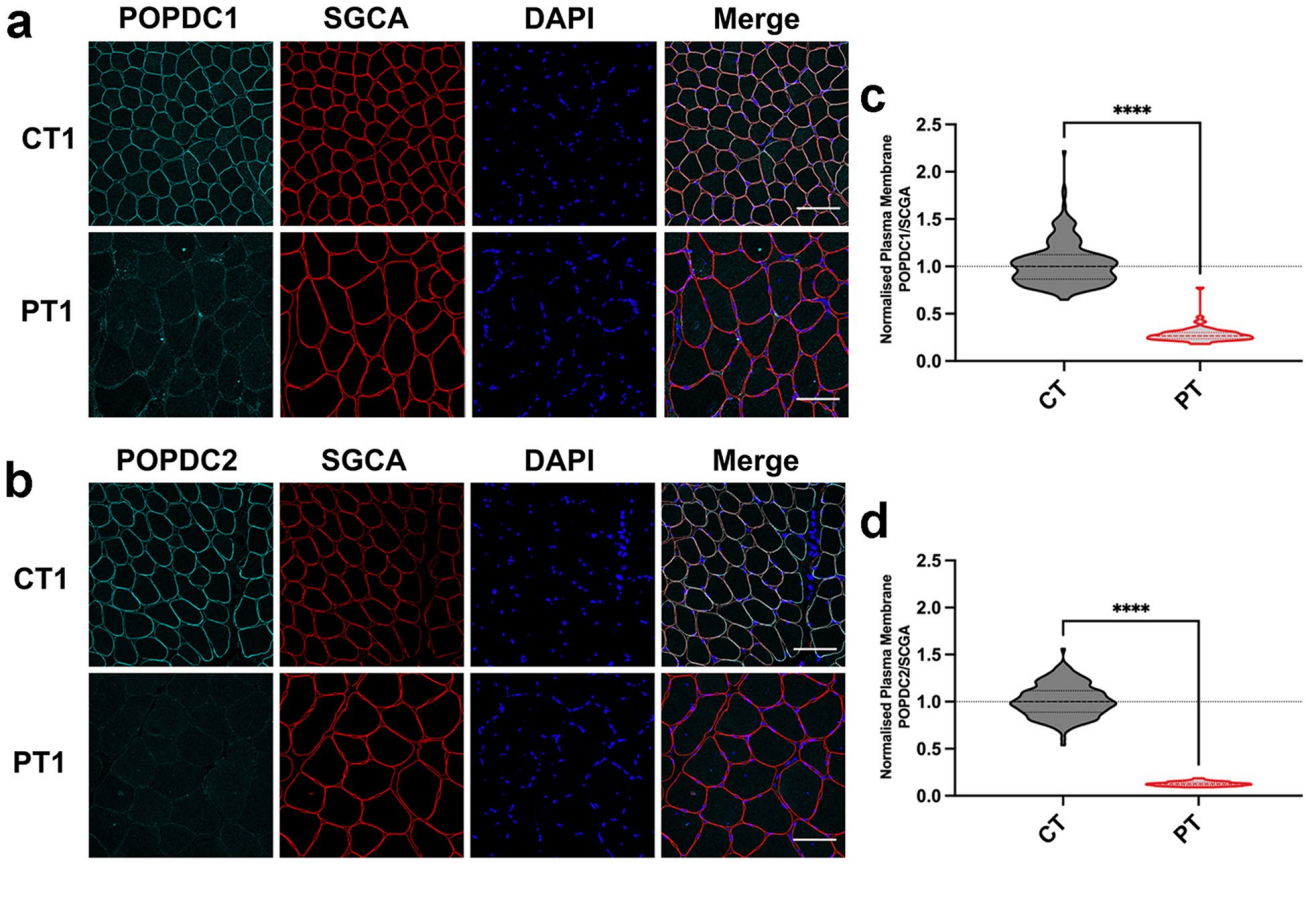
The expression of POPDC1 and POPDC2 is greatly reduced at the sarcolemma of skeletal muscle fibers expressing *POPDC1* p.Q153X. **a** and **b** Transverse sections of skeletal muscle biopsies from a patient (PT) carrying the *POPDC1* p.Q153X variant in homozygosity and a matched control (CT) were stained for **a** POPDC1 or **b** POPDC2, along with SGCA as a sarcolemma marker. Scale bar: 100 μm. **c** and **d** The expression levels of **c** POPDC1 and **d** POPDC2 in the sarcolemma normalized to SGCA, were quantified in individual fibers. The number of sections (sec), images (img) and fibers (fib) analyzed per group are as follows: CT: POPDC1—1 sec, 4 img, 238 fib; POPDC2—1 sec, 4 img, 163 fib. PT: POPDC1—1 sec, 3 img, 65 fib; POPDC2—1 sec, 3 img, 70 fib. The median POPDC/SGCA-level in each control biopsy was set to 1. Dashed lines indicate the normalized median and interquartile range. Data were analyzed using Mann–Whitney test; ****p < 0.0001

A recently reported nonsense mutation in *BVES* (c.457 > T, p.Q153X) is associated with early onset sinus bradycardia and AV-block and high serum CK levels without clinical signs for LGMD [15]. This mutation is predicted to lead to a truncation of POPDC1 within the Popeye domain, removing the cAMP binding domain and cytoplasmic C-terminal tail. A qualitative reduction in POPDC1 expression at the sarcolemma was reported in the affected patient, however no analysis of POPDC2 expression was performed [15]. We have now quantified the changes in expression levels of POPDC1 and POPDC2 in the muscle fibers contained in biopsies from the index patient, along with a matched control (Fig. 3a, b). The median SGCA-normalized POPDC1 intensity at the sarcolemma was around 75% lower (*p* < 0.0001) in the patient (0.266, 95% CI 0.250, 0.282; n = 65) compared to the matched control (1.000, 95% CI 0.962, 1.026; n = 238). The normalized POPDC2 sarcolemmal level was even further reduced, by 88% (*p* < 0.0001), between the patient (0.124, 95% CI 0.116, 0.130; n = 70) and the control (1.000, 95% CI 0.970, 1.038; n = 163) (Fig. 3c, d). The absolute changes in POPDC1 and POPDC2 staining intensity at the sarcolemma were similar (Additional file 1: Fig. S2a, d). A small decrease in POPDC1 and a moderate increase in POPDC2 were seen in the cytoplasmic levels (Additional file 1: Fig. S2b, e). This led to a highly consistent reduction in the enrichment of POPDC1 and POPDC2 at the sarcolemma of the patient’s muscle fibers, with minimal variability (Additional file 1: Fig. S2c, f). It was also noted that there was a greater than 2.5-fold increase (*p* < 0.0001) in the cross-sectional area of muscle fibers between the control (2031 μm^2^, 95% CI 1968, 2079, n = 401) and patient biopsies (5283 μm^2^, 95% CI 4707, 5952, n = 135) (Additional file 1: Fig. S2g).

### Muscle fibers of Popdc2^W188X/W188X^ mutants show a loss of POPDC1 and POPDC2 at the sarcolemma

**Fig. 4:**
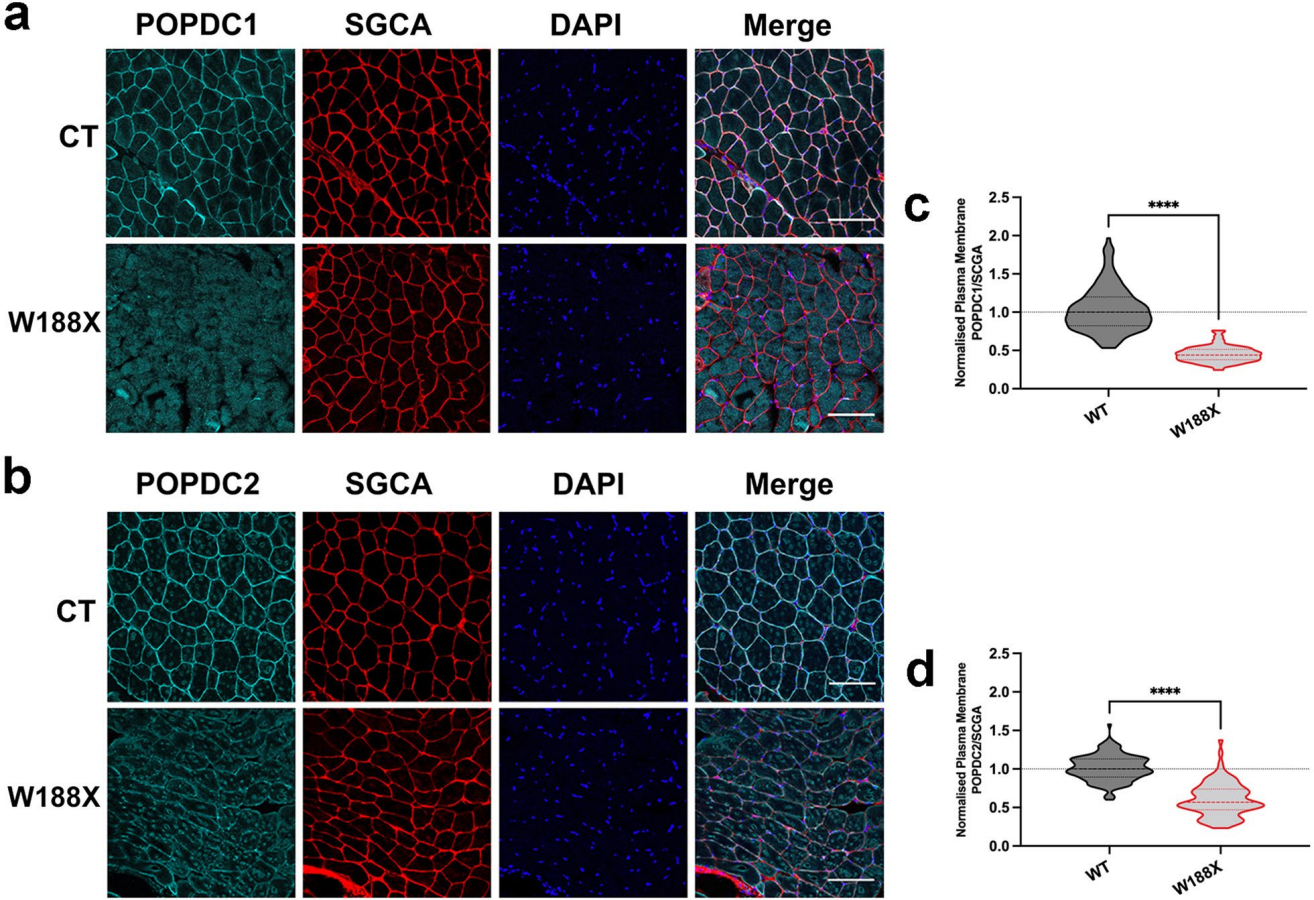
Intermediate reduction of POPDC1 and POPDC2 expression at the sarcolemma of skeletal muscle fibers of a homozygous *Popdc2*^W188X/W188X^ knockin mouse. **a** and **b** Transverse sections of the *m. gastrocnemius* of a 3-month-old homozygous *Popdc2*^W188X/W188X^ mouse mutant (W188X) and a wild-type control (WT) were stained for (A) POPDC1 or (B) POPDC2, along with SGCA as a sarcolemma marker. Scale bar: 100 μm. **c** and **d** The expression levels of **c** POPDC1 and **d** POPDC2 in the sarcolemma, normalized to SGCA, were quantified in individual fibers. The number of sections (sec), images (img) and fibers (fib) analyzed per group are as follows: WT: POPDC1—1 sec, 3 img, 164 fib; POPDC2—1 sec, 3 img, 143 fib. W188X: POPDC1—1 sec, 4 img, 95 fib; POPDC2—1 sec, 4 img, 93 fib. The median POPDC/SGCA-level in each control biopsy was set to 1. Dashed lines indicate the normalized median and interquartile range. The control and homozygous mutant pairs were compared using a Mann–Whitney test; **** *p* < 0.0001

A heterozygous *POPDC2* (c.563G > A, p.W188X) mutation was previously reported in patients displaying AV-block [37]. No investigations into changes in the sarcolemmal expression of POPDC1 or the POPDC2 in skeletal muscle or heart tissue were performed due to a lack of biopsy material. We have created a homozygous *Popdc2*^W188X/W188X^ knockin mouse [37]. The *gastrocnemius* was dissected from mutant and wild-type (WT) mice and sections were stained for POPDC1 (Fig. 4a) or POPDC2 (Fig. 4b) along with SGCA, and the differences in staining compared. A 56% reduction (*p* < 0.0001) was seen in the median SGCA-normalized POPDC1 intensity at the sarcolemma of each muscle fiber of the mutant (0.440, 95% CI 0.416, 0.467, n = 95) and WT (1.000, 95% CI 0.939, 1.043; n = 164) (Fig. 4c). POPDC2 at the sarcolemma was reduced by 43% (*p* < 0.0001) in the *Popdc2*^W188X/W188X^ mutant (0.471, 95% CI 0.523, 0.661; n = 93) and WT (1.000, 0.976, 1.050; n = 143) (Fig. 4d). The absolute change in POPDC1 and POPDC2 at the sarcolemma was similar (Additional file 1: Fig. S3a, d) while only very mild changes in cytoplasmic levels of both POPDC isoforms were seen (Additional file 1: Fig. S3b, e). This resulted in a lowering of the excess of POPDC1 and POPDC2 at the sarcolemma compared to the cytoplasm, with minimal variability (Additional file 1: Fig. S3c, f). No major aberrations in fiber morphology were observed (Fig. 4a, b), although a 10% reduction in the average fiber cross-sectional area (*p* = 0.023) was seen in the mutant compared to wild type (Additional file 1: Fig. S3g).

### Different POPDC mutations have a variable impact on sarcolemmal expression of POPDC1 and POPDC2

**Fig. 5:**
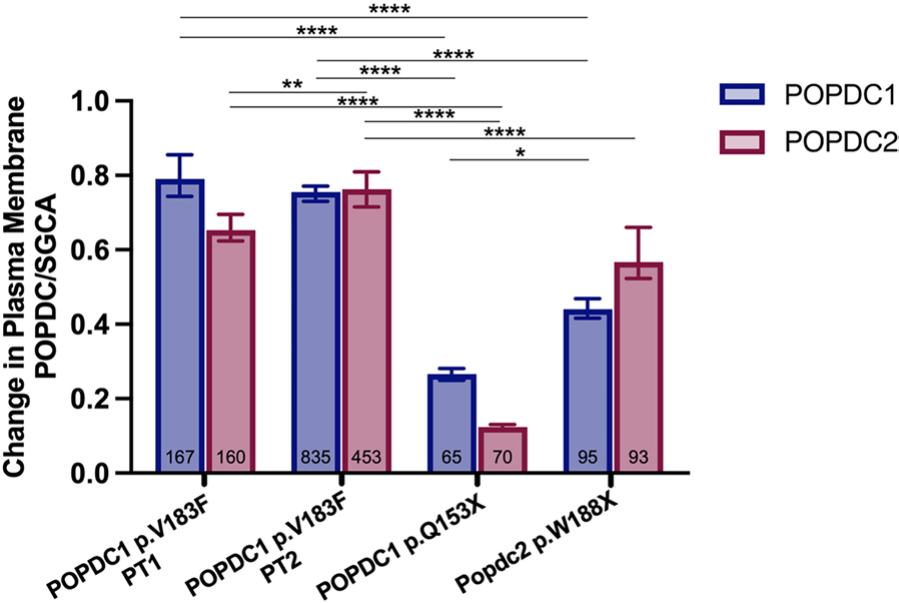
POPDC mutations have varying impacts on POPDC1 and POPDC2 sarcolemmal expression in skeletal muscle. Comparison of the fold change in POPDC1 and POPDC2 expression, normalized to SGCA, in the sarcolemma of skeletal muscle fibers from biopsy material of patients carrying the *POPDC1* p.V183F and *POPDC1* p.Q153X variants and a homozygous *Popdc2* p.W188X knock-in mouse compared to matched controls or wild-type mouse. The total number of fibers analyzed are shown at the base of each bar. Data is displayed as median ± 95% CI. POPDC1 and POPDC2 values were compared using Kruskal–Wallis followed by Dunn’s multiple comparisons test; * *p* < 0.05; ** *p* < 0.01; **** *p* < 0.0001

While all of the here studied mutations led to a reduction in the sarcolemmal expression level of POPDC1 and POPDC2, the effects were variable (Fig. 5). Comparing the changes between each biopsy and its respective matched controls showed that the reduction in POPDC1 and POPDC2 levels at the sarcolemma in the case of the two patients carrying the POPDC1 p.V183F variant was significantly less than that seen in the patient possessing the POPDC1 p.Q153X mutation (*p* < 0.0001; Fig. 5). While there was no difference in the effect on POPDC1 across the two V183F patients (*p* = 0.078; Fig. 5), the reduction in POPDC2 was around 10% greater in PT1 (p = 0.0030; Fig. 5). The effect in the *Popdc2*^W188X/W188X^ mouse was less severe than in the patient expressing POPDC1 p.Q153X with respect to the loss of POPDC1 (*p* = 0.016; Fig. 5) and POPDC2 (*p* < 0.0001; Fig. 5). The effect on POPDC1 expression was however greater in the *Popdc2*^W188X/W188X^ mouse mutant than in both patients expressing POPDC1 p.V183F as well as for POPDC2 in case of PT2 carrying the POPDC1 p.V183F variant (*p* < 0.0001; Fig. 5). No difference was seen between the impact on POPDC2 sarcolemmal expression in the case of PT1 carrying the POPDC1 p.V183F mutation and the *Popdc2*^W188X/W188X^ mouse mutant (*p* = 0.32; Fig. 5).

### The plasma membrane expression and trafficking of POPDC1 and POPDC2 is dependent on each other

**Fig. 6:**
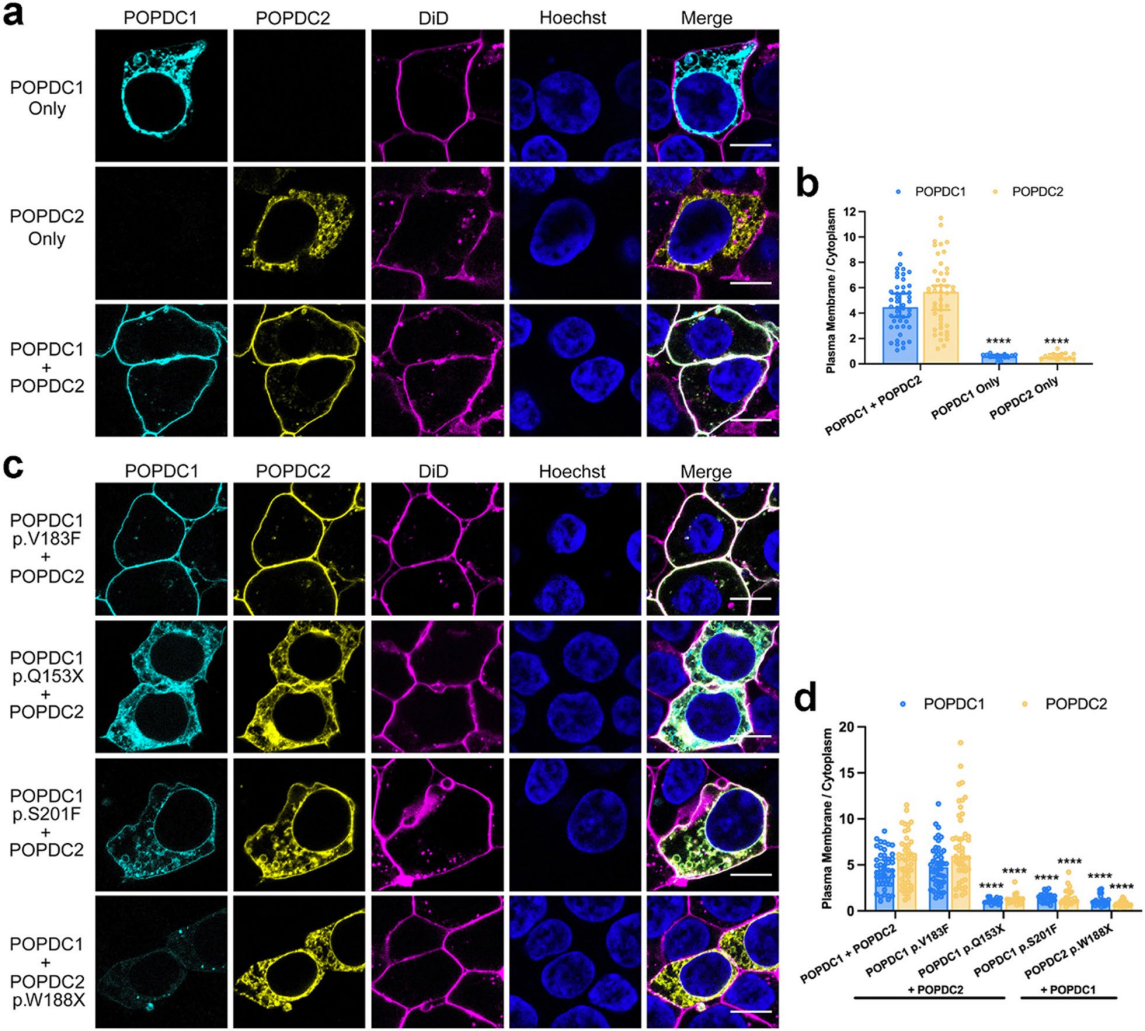
Co-expression of POPDC1 and POPDC2 is required for plasma membrane localization in HEK293 cells. **a** POPDC1-ECFP, POPDC2-EYFP, or both were transiently expressed in HEK293 cells. The plasma membrane was marked using DiD. Scale bar: 10 μm. **b** Bar graph of the ratio of plasma membrane to cytoplasm expression of POPDC1 or POPDC2 (POPDC1-ECFP: n = 15, POPDC2-EYFP: n = 15, POPDC1-ECFP + POPDC2-EYP: n = 46; min., N ≥ 2). Bars show median ± 95% CI. The groups were compared using a Mann–Whitney test; ****p < 0.0001. **c** POPDC1 V183F-ECFP, Q153X-ECFP and S201F-ECFP and POPDC2 W188X-EYFP constructs were co-expressed with the appropriate wild-type POPDC partner in HEK293 cells. The plasma membrane was marked using DiD. Scale bar: 10 μm. **d** Bar graph of the ratio of plasma membrane to cytoplasm expression of POPDC1 or POPDC2 in the presence of the different POPDC1 and POPDC2 mutant proteins (WT n = 46, V183F n = 47, Q153X n = 17, S201F n = 22, W188X n = 24, min., N ≥ 2). Identical data are shown in **b** and **d** for the expression levels after co-transfection of both wild-type constructs. Bars show median ± 95% CI. Groups were compared using Kruskal–Wallis followed by Dunn’s test using the wild-type pair for comparison; ****p < 0.0001

The above findings, as well of those from other previously reported patients [11, 19, 40], suggest that a mutation in POPDC1 can alter the subcellular expression pattern of POPDC2, and vice versa, in skeletal muscle fibers. To investigate if the membrane expression of POPDC1 and POPDC2 is indeed dependent on each other, HEK293 cells were transiently transfected with either POPDC1 or POPDC2 possessing C-terminal ECFP and EYFP tags, respectively (Fig. 6a). Individual cells were segmented into cytoplasm and plasma membrane compartments using the lipophilic dye DiD to mark the plasma membrane and Hoechst-33342 to demarcate the nucleus. The extent of the plasma membrane localization of each protein was quantified by determining the relative level of each protein at the plasma membrane compared to the cytoplasm. The median level of localization of each protein across cells when singly expressed, or when co-expressed with the other POPDC isoform, was compared. When singly expressed, POPDC1 (0.565, 95% CI 0.502, 0.713; n = 15) and POPDC2 (0.506, 95% CI 0.403, 0.773, n = 15) were almost half as concentrated in the plasma membrane compared to the cytoplasm (Fig. 6b). However, when co-expressed, POPDC1 (4.461, 95% CI 3.692, 5.536; n = 46) and POPDC2 (5.666, 95% CI 4.254, 6.160, n = 46) were both effectively localized to the plasma membrane at levels significantly above the single expression conditions (*p* < 0.0001; Fig. 6b). No significant difference in cytoplasmic levels of POPDC1 and POPDC2 between the two groups was seen (Additional file 1: Fig. S4a). However, it was found that POPDC1 and POPDC2 plasma membrane expression when solely expressed was around 30% and 10% of the level observed in the co-expression system, respectively (*p* < 0.0001; Additional file 1: Fig. S4b).

The effect of a set of clinically identified POPDC mutations on the subcellular expression of POPDC1 and POPDC2 in HEK293 cells was then investigated. The POPDC1 p.V183F and p.Q153X mutations were introduced into the POPDC1-ECFP construct. Additionally, a POPDC1 p.S201F mutant was tested, having previously been reported to cause a significant loss in the plasma membrane expression of POPDC1 and POPDC2 in the muscle fibers of patients [40]. The POPDC2 p.W188X mutation was introduced into the POPDC2-EYFP construct. Each mutant construct was co-transfected with its corresponding wild-type partner and the change in the median plasma membrane localization of each protein, compared to the double wild-type expression, was determined. A significant reduction in the plasma membrane localization of POPDC1 was seen in the presence of POPDC1 p.Q153X (0.967, 95% CI 0.916, 1.243; n = 17), POPDC1 p.S201F (1.520, 95% CI 1.012, 1.735; n = 22), and POPDC2 p.W188X (0.983, 95% CI 0.830, 1.153; n = 24) compared to the wild-type pair (all *p* < 0.0001; Fig. 6c, d). Likewise, significant drops in the plasma membrane enrichment of POPDC2 compared to the wild-type pair was seen for POPDC1 p.Q153X (1.239, 95% CI 0.973, 1.369; n = 17), p.S201F (1.202, 95% CI 0.911, 2.078; n = 22), and POPDC2 p.W188X (0.682, 95% CI 0.573, 0.872; n = 24) (all *p* < 0.0001; Fig. 6c, d). The changes in the expression of the constructs in the cytoplasm and plasma membrane relative to the wild-type pair, which caused the observed changes in the level of plasma membrane localization, were as follows. The POPDC1 p.Q153X mutation led to an increased accumulation of POPDC1 (2.960, 95% CI 1.062, 5.464; *p* = 0.015) and POPDC2 (2.300, 95% CI 1.255, 4.554; *p* = 0.042) in the cytoplasm, while POPDC2 p.W188X led to an increase in the intracellular localization of the mutant POPDC2 protein (5.817, 95% CI 3.345, 7.374; *p* < 0.0001). The POPDC1 p.V183F mutant resulted in a mild drop in POPDC2 within the cytoplasm (0.5812, 95% CI 0.428, 0.806; *p* = 0.029). The cytoplasmic level of both isoforms was unchanged in the case of POPDC1 p.S201F (Additional file 1: Fig. S4c). The concentration of both proteins at the plasma membrane was comparable to wild type in the presence of the POPDC1 p.V183F mutant. POPDC1 p.Q153X led to mild reductions in the POPDC2 construct (0.618, 95% CI 0.270, 0.925; *p* = 0.35). The POPDC1 p.S201F led to major reduction of the mutant POPDC1 (0.233, 95% CI 0.158, 0.437) and its POPDC2 partner (0.276, 95% CI 0.184, 0.540) (both *p* < 0.0001). Additionally, POPDC1 when co-expressed with POPDC2 p.W188X was reduced at the plasma membrane (0.344, 95% CI 0.207, 0.656; *p* < 0.0001) (Additional file 1: Fig. S4d).

### Direct interaction of POPDC1 and POPDC2

**Fig. 7:**
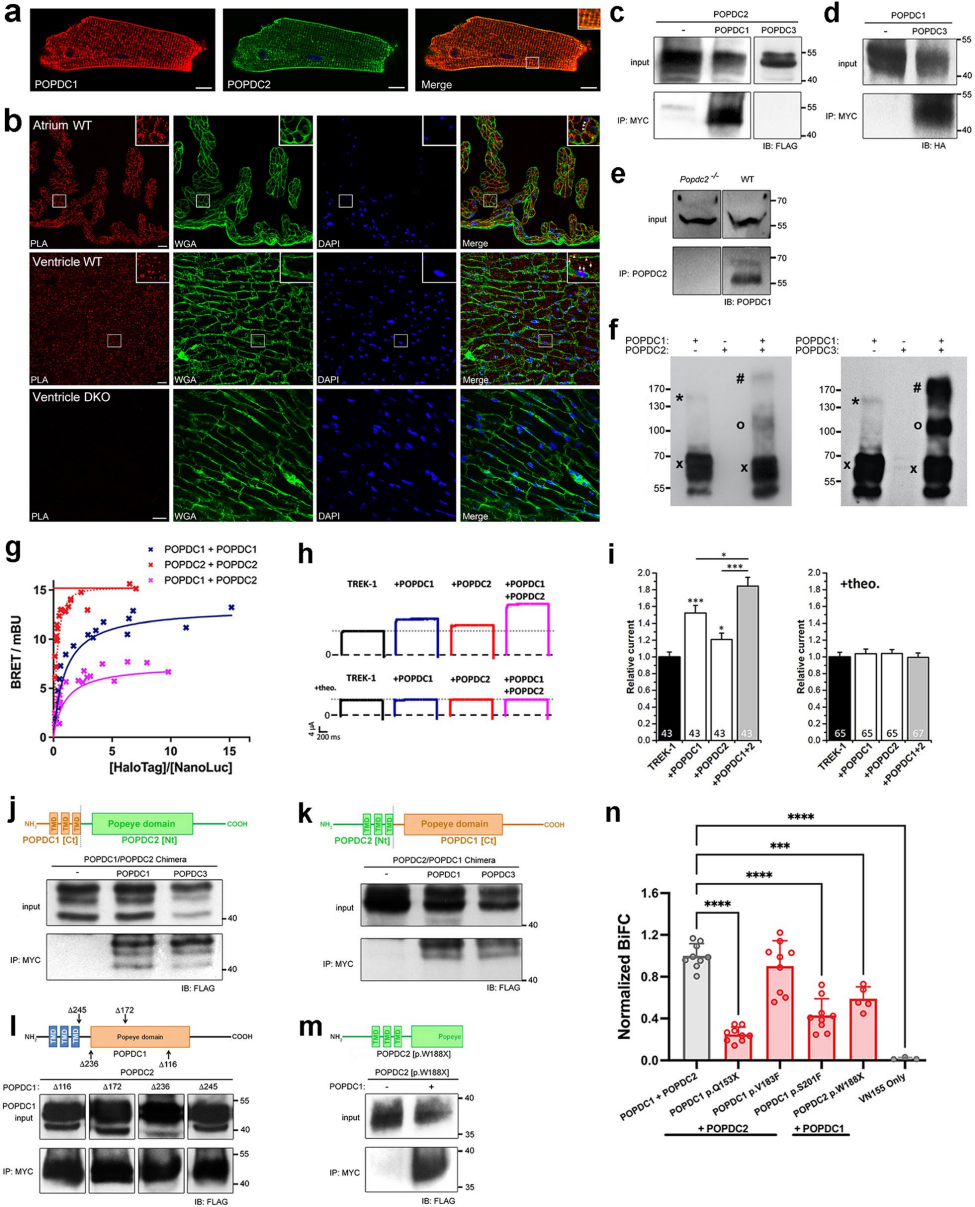
POPDC1 and POPDC2 undergo heteromeric complex formation. **a** Adult mouse ventricular cardiomyocytes immunostained for POPDC1 (red) and POPDC2 (green). **b** PLA of POPDC1 and POPDC2 in transverse sections of right atrium and left ventricle from wild-type and *Popdc1*/*Popdc2* knockout mice. Sections were counterstained with WGA (green) and DAPI. **c** and **d** Co-precipitation of **c** POPDC2-FLAG alone or together with POPDC1-MYC or POPDC3-MYC, respectively, or **d** POPDC1-HA alone or together with POPDC3-MYC. **e** Co-precipitation of ventricular tissue lysates of *Popdc2* null mutant and wild-type mice. **f** Western blot of lysates from COS-7 cells expressing POPDC1-CFP and/or POPDC2-FLAG (left), or POPDC1-CFP and/or POPDC3-MYC (right). (x) monomer, (*) homodimer, (o) heterodimer (#) heterotetramer. **g** Quantitative Type-1 BRET saturation curves of POPDC1 and POPDC2 homo- and heteromeric complexes (N ≥ 2). **h** and **i** TREK-1 current in *Xenopus* oocytes expressing TREK-1 alone or together with POPDC1, POPDC2, or both. **h** Examples of TREK-1 current in response to a voltage jump from − 80 to 40 mV and **i** relative current amplitudes without or with theophylline (+ theo). Number of oocytes are given in each graph. Data are presented as mean ± SEM. *p < 0.05; ***p < 0.001. **j** and **k** Chimeric constructs containing **j** the N-terminal and transmembrane domains of POPDC1 and the cytoplasmic region of POPDC2 or **k** N-terminal and transmembrane domains of POPDC2 and the cytoplasmic region of POPDC1 were co-transfected with POPDC1 or POPDC3, respectively and subjected to co-precipitation analysis. **l** Truncations were introduced into POPDC1-MYC and subjected to co-precipitation analysis in COS-7 cells after co-transfection with POPDC2-FLAG. **m** A POPDC2-FLAG construct truncated to residue W188 was subjected to co-precipitation analysis after co-expression with POPDC1-MYC. **n** BiFC signal after co-expression of wild-type POPDC1 and POPDC2, or of POPDC1 p.V183F, p.Q153X, p.S201F and POPDC2 p.W188X mutants in HEK293 cells. POPDC1-VN155 + POPDC2-VN155 was used as a negative control. ***p < 0.001; ****p < 0.0001

While POPDC1 has previously been reported to form homodimers [23, 26], the above results led us to search for evidence of a direct interaction between POPDC1 and POPDC2. Both POPDC1 and POPDC2 are prominently expressed in cardiac and skeletal muscle [2, 35] and immunostaining of isolated ventricular cardiac myocytes revealed overlapping expression domains for both isoforms (Fig. 7a). To identify any interactions between POPDC1 and POPDC2 in their native environment, a PLA was carried out using sections from mouse atrium and ventricle from wild-type mice, with ventricular tissue of *Popdc1*^−/−^/*Popdc2*^−/−^ mutants serving as negative control (Fig. 7b). PLA signals were observed in atrial and ventricular sections of wild-type hearts, whereas no signal was present in sections of *Popdc1*^−/−^/*Popdc2*^−/−^ mutants. While PLA signals were observed in cardiac myocytes of both chambers, the subcellular localizations differed between atrial and ventricular myocytes. In atrial myocytes, PLA signals were mostly localized at the sarcolemma, whereas in ventricular myocytes signals were found at the sarcolemma and within the cell boundaries. POPDC1 and POPDC2 have both been shown to reside in the sarcolemma and in the t-tubules of cardiomyocytes [1], with the higher level of t-tubules present in ventricular cardiomyocytes [28] likely contributing to the observed chamber-specific differences. This shows that POPDC1 and POPDC2 form complexes at the sarcolemma in cardiomyocytes. To confirm the interaction of POPDC1 and POPDC2, POPDC2 possessing a C-terminal FLAG-tag was co-expressed with a POPDC1-MYC construct in COS-7 cells. In addition, POPDC2-FLAG was also co-expressed with POPDC3-MYC. Cell lysates were precipitated with a MYC-tag antibody and subjected to Western blot analysis using FLAG-tag antibody. POPDC1 was found to specifically co-precipitate with POPDC2, but this was not the case for POPDC3 (Fig. 7c). Performing the same experiment with POPDC1 carrying a C-terminal HA-tag and POPDC3 with a MYC-tag demonstrated that POPDC3 can be co-precipitated with POPDC1 (Fig. 7d). This suggests that POPDC1 undergoes complex formation with POPDC2 and POPDC3, but no interaction is detectable between POPDC2 and POPDC3. The interaction of POPDC1 and POPDC2 was also further demonstrated to occur in native tissue by co-immunoprecipitation of POPDC1 from mouse heart lysates using a POPDC2 antibody (Fig. 7e). The co-precipitation of POPDC1 did not occur when lysates were used from the hearts of *Popdc2* null mutant mice.

When lysates of COS-7 cells expressing POPDC1 and POPDC2 were examined using SDS-PAGE followed by a Western blot, evidence of POPDC1 forming hetero-oligomers with POPDC2 was seen. Both POPDC1 and POPDC2 isoforms have very similar molecular weights (POPDC1: 41.5 kDa, POPDC2: 40.5 kDa). Therefore, POPDC1 was tagged at the C-terminal with CFP (29 kDa), while POPDC2 was fused with FLAG (1 kDa), to enable differentiation of homo- (expected: 140.5 kDa) and heterodimers (expected: 111.5 kDa) based on their molecular weight on the blot. Use of an anti-CFP antibody showed a differing pattern of bands when POPDC1 was expressed alone compared to co-expression with POPDC2. The majority of POPDC1 was in a monomeric state (multiple bands of approx. 70 kDa) in both groups, as would be expected given the presence of SDS. However, weaker bands corresponding to homodimers of POPDC1 (approx. 140 kDa), as well as those matching the expected molecular weight of POPDC1–POPDC2 heterodimers (approx. 110 kDa) and heterotetramers (approx. 220 kDa) were also seen, despite the denaturing conditions during electrophoresis (Fig. 7f). Similar results were also seen when POPDC1 was co-expressed with POPDC3.

To investigate the stoichiometries of POPDC1–POPDC2 complexes within a cellular environment, a type-1 quantitative BRET (qBRET) assay was employed, following a previously reported protocol [13]. The NanoBRET platform [30] was utilized in the assay by tagging POPDC1 and POPDC2 at the C-terminus with NanoLuc luciferase (NL) or HaloTag (HT) fusion tags. These constructs were co-expressed in HEK293 cells at varying expression ratios, but at constant total expression levels (Additional file 1: Fig. S5) and the relationship to the BRET signal analyzed. Firstly, POPDC1-NL and POPDC1-HT were co-transfected to try and identify if homomeric interactions were present as previously reported [23, 26]. A clear hyperbolic BRET saturation curve indicative of a dimer was apparent (Fig. 7g). A rapidly saturating BRET curve was observed in the case of co-expression of POPDC2-NL and POPDC2-HT. Rapid saturation is a feature of higher order complexes, although such curve shapes make accurate determination of complex stoichiometry via type-1 qBRET studies difficult [13]. When POPDC1-HT and POPDC2-NL were co-expressed, the BRET saturation curve produced fitted to a dimer model. This suggests that the major POPDC1–POPDC2 complex is a dimer. Such heterodimers would have to compete against the tendency of POPDC1, and likely POPDC2, to form homodimers, which suggests that the heteromeric-interaction of POPDC1 and POPDC2 is favored.

We have previously shown that co-expression of POPDC1 or POPDC2 with the 2-pore domain potassium channel TREK-1 in *Xenopus laevis* oocytes leads to an increase in the outward K^+^ current compared to expression of TREK-1 alone [14]. This was attributed to a direct, cAMP-sensitive interaction between POPDC proteins and TREK-1. Incubation of the cells with 8-Br-cAMP, or the phosphodiesterase inhibitor theophylline, abolishes the increase in current in the presence of POPDC1 or POPDC2, respectively [14, 40]. To test if the formation of heteromeric POPDC1–POPDC2 complexes could modulate TREK1 current, one or both POPDC isoforms were expressed in *Xenopus laevis* oocytes alongside TREK-1 and two electrode voltage-clamp measurements used to determine the outward K^+^ current from cells. As expected, co-expression of POPDC1 or POPDC2 with TREK-1 led to a significant increase in TREK-1 current compared to TREK-1 alone (Fig. 7h, i). Furthermore, co-expression of POPDC1 and POPDC2 led to a significant additional increase in TREK-1 current above the levels observed with POPDC1 or POPDC2 alone. When the *Xenopus* oocytes were incubated with theophylline, the increase in TREK1 current after expression of POPDC1 and/or POPDC2 returned to baseline levels as previously reported [14, 40].

To help identify the domains responsible for the POPDC1–POPDC2 and POPDC1–POPDC3 interactions, two FLAG-tagged chimeras were constructed, consisting of the N-terminal and transmembrane domains of POPDC1 and the cytoplasmic region (including the Popeye domain) of POPDC2 and the inverse configuration (Fig. 7j, k). Each chimera was co-expressed in COS-7 cells with MYC-tagged POPDC1 or POPDC3. Precipitation of cell lysates using MYC-antibody led to co-precipitation of both chimeras in all cases.

To further map the sites in POPDC1, which mediate the interaction between POPDC1 and POPDC2, co-immunoprecipitation experiments in COS-7 cells were repeated in the presence of various C-terminal truncations of POPDC1 (Fig. 7l). The truncation mutants were C-terminally tagged with a MYC epitope and full-length POPDC2 with FLAG. Truncations were positioned to delete the C-terminal tail and end of the Popeye domain (Δ116), the C-terminal tail and half of the Popeye domain (Δ172), the C-terminal tail and the entire Popeye domain (Δ236) and the entire cytoplasmic region of POPDC1 (Δ245). After co-expression of these constructs with POPDC2, it was found that co-immunoprecipitation of POPDC2-FLAG was possible with all the truncation mutants, suggesting that the extracellular N-terminal region and transmembrane domains of POPDC1 were sufficient to form an interaction with POPDC2. A similar conclusion can probably be drawn for POPDC2, as the POPDC2 W188X mutant protein, which lacks the carboxy-terminal half of the Popeye domain and the carboxy terminus still retains the ability to interact with POPDC1 (Fig. 7m). These results, and behavior of the chimeric constructs, show that POPDC1–POPDC2 and POPDC1–POPDC3 interactions, occur at both the N-terminal/transmembrane domains and cytoplasmic portions of the proteins.

It was shown above that the POPDC1 p.Q153X, p.S201F, and POPDC2 p.W188X mutations led to mislocalization of POPDC1 and POPDC2 in HEK293 cells, as well as in skeletal muscle, while the POPDC1 p.V183F mutation had no effect in HEK293 cells and led to only mild changes in POPDC expression patterns in tissue. To investigate if a change in the interaction between POPDC1 and POPDC2 was responsible for this effect, a bimolecular fluorescence complementation (BiFC) assay was conducted (Fig. 7n). POPDC1 and POPDC2 wild-type and mutant constructs were tagged at the C-terminal with the split Venus domains VC155 and VN155, respectively, and expressed in HEK293 cells. Interactions between VC155 and VN155 lead to reconstitution of the Venus fluorophore. POPDC1-VN155 expressed with POPDC2-VN155 was utilized as a negative control, while mRFP was co-transfected into the cells to act as an internal transfection control to which BiFC signals were normalized. The mRFP signal was also used to define areas containing transfected cells within confocal microscopy images (approx. 50–100 cells per image) from which the BiFC signal was measured (Additional file 1: Fig. S6). As expected, wild-type POPDC1-VC155 and POPDC2-VC155 yielded a strong BiFC signal, which was set to equal 1, providing further evidence for the existence of POPDC1–POPDC2 complexes. No difference in the BiFC signal from POPDC1 p.V183F + POPDC2 compared to the wild-type pair was seen (n = 9, *p* = 0.54). However, a significant drop in the BiFC signal relative to the wild-type pair was observed in the presence of the POPDC1 p.Q153X (0.248, 95% CI 0.195, 0.301; n = 9), p.S201F (0.430, 95% CI 0.307, 0.553; n = 9), and POPDC2 p.W188X (0.590, 95% CI 0.449, 0.730; n = 5) variants (all *p* < 0.0001). However, a detectable BiFC signal greatly above background was observed in all groups suggesting the POPDC1–POPDC2 interaction was not fully abolished.

### POPDC1 and POPDC2 may interact through a conserved interface in the αC-helix of the Popeye domain

**Fig. 8:**
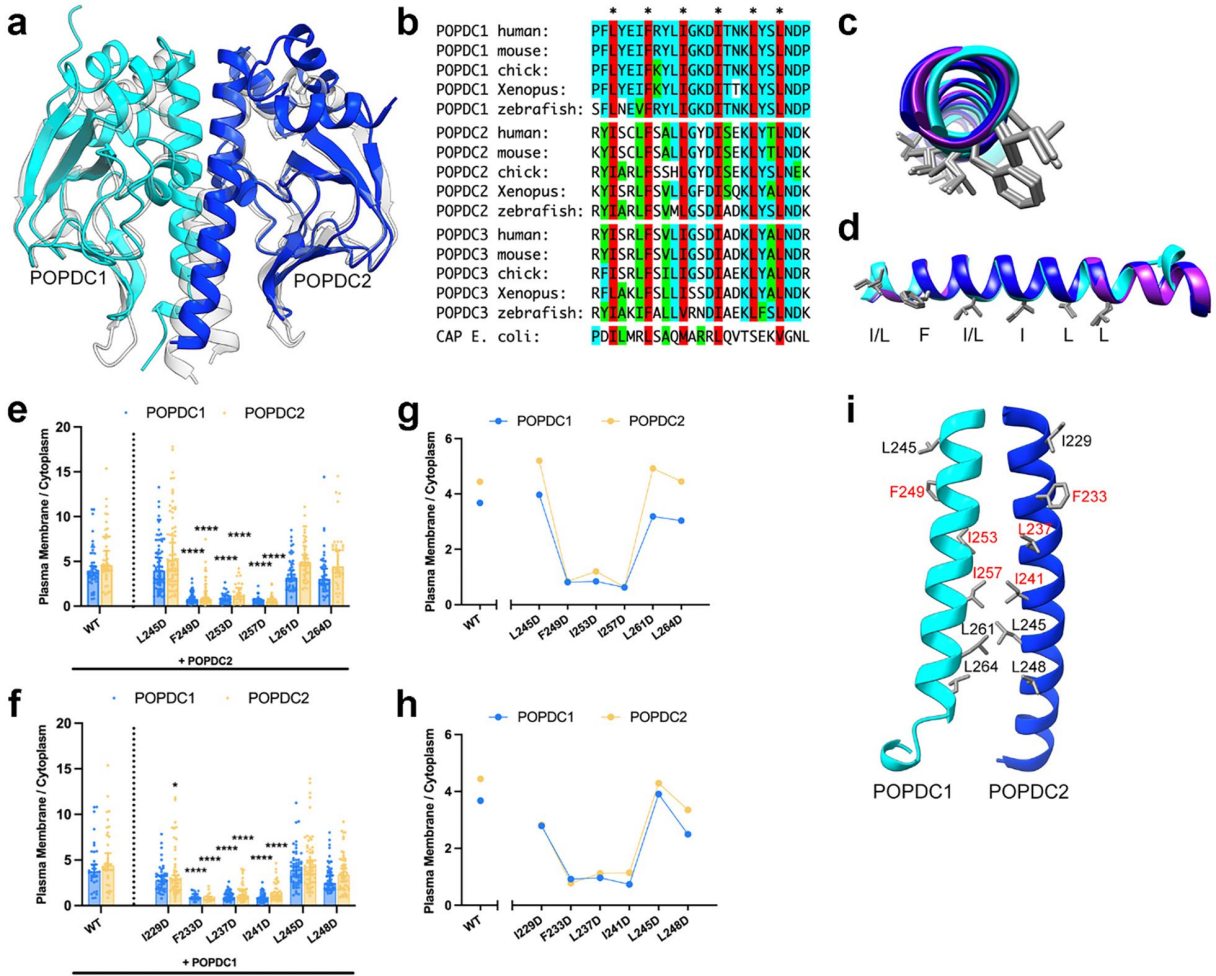
The αC-helix of the Popeye domain mediates heteromeric complex formation between POPDC1 and POPDC2. **a** A model of heteromeric complex formation of the Popeye domains of POPDC1 (cyan) and POPDC2 (blue) using the structure of a CAP dimer (translucent; PDB: 1G6N) as a template. **b** Sequence alignment of the αC-helix of POPDC1, POPDC2 and POPDC3 from multiple vertebrate species and CAP protein from E. coli. A set of highly conserved hydrophobic amino acids are highlighted in red. **c** and **d** Overlay of the predicted αC-helical structures of POPDC1 (cyan), POPDC2 (blue) and POPDC3 (purple) with the side chains of the highly conserved hydrophobic residues. **c** Amino terminal and **d** side view. **e** and **f** The ratio of plasma membrane to cytoplasm expression levels of POPDC1-ECFP and POPDC2-EYFP in HEK293 cells, where either **e** POPDC1-ECFP or **f** POPDC2-EYFP was subjected to site-directed mutagenesis to introduce aspartic acid in place of a conserved hydrophobic residue within the αC-helix sequence. Total number of cells analyzed: POPDC1: L245D n = 71, F249D n = 56, I253D n = 27, I257D n = 40, L261D n = 47, L264D n = 42; POPDC2: I229D n = 48, F233D n = 20, L237D n = 45, I241D n = 42, L245D n = 63, L248D n = 56, N ≥ 2. Min. 2 transfections per group. Bars show median ± 95% CI. Groups were compared using Kruskal–Wallis followed by Dunn’s test using the wild-type pair as a comparison; **p < 0.01, ****p < 0.0001. **g** and **h** Relationship between plasma membrane versus cytoplasm expression and each mutation in **g** POPDC1 and **h** POPDC2. **i** The predicted αC-helical Popeye domain interface between POPDC1 and POPDC2. Hydrophobic residues whose mutation to aspartic acid led to severely impaired plasma membrane localization of both POPDC proteins are labelled in red

Having demonstrated that POPDC1 and POPDC2 interact through both their N-terminal/ transmembrane and cytoplasmic regions, we focused on the role of the Popeye domain, which was previously reported to be involved in POPDC1 homomeric interactions [23, 26]. The cAMP binding domain of the prokaryotic cAMP-binding transcriptional regulator catabolite activator protein (CAP) shows the highest sequence similarity to the Popeye domain [43] and has therefore been used previously as a template for producing homology models of the Popeye domain [14]. CAP protein monomers dimerize through an α-helix at the C-terminal end of their cyclic nucleotide binding domain (CNBD), known as the C-helix, via a set of hydrophobic residues [31] (Additional file 1: Fig. S7a, b). As well as forming an interface between the CAP subunits, the C-helix also forms contacts with cAMP upon binding (Additional file 1: Fig. S7c) [34, 42]. The Popeye domains of POPDC1 and POPDC2 are predicted to be highly similar in structure to the CNBD of CAP [14], and the protein structure of the CAP dimer (PDB: 1G6N [31]) was utilized as a template to model the POPDC1–POPDC2 heteromeric complex (Fig. 8a). An α-helix is predicted to form at the C-terminal end of the Popeye domain and is referred to as the αC-helix in reference to the structures of PKA and other cAMP effector proteins [36, 46]. The model of the Popeye domain dimer possesses an interface between each αC-helix, analogous to the C-helix interface in CAP. Alignment of the amino acid sequences of the αC-helices of vertebrate POPDC1, POPDC2 and POPDC3, as well as the C-helix of CAP, reveals a high level of sequence conservation and in particular the invariant presence of a series of hydrophobic residues in each POPDC isoform, which, with the exception of one residue, were also present in the C-helix of CAP (Fig. 8b). Some of these residues are known to be involved in CAP dimerization (Additional file 1: Fig. S7b) [31, 34]. These hydrophobic residues show very strong structural alignment across the predicted structures of the αC-helix in POPDC1, POPDC2, and POPDC3 (Fig. 8c, d). The results shown above suggest that if normal POPDC1–POPDC2 interactions are disrupted, or absent, then the subcellular expression of both isoforms is altered. To determine if the CAP-aligned, highly conserved hydrophobic residues within the αC-helices of POPDC1 and POPDC2 are involved in complex formation, POPDC1-ECFP and POPDC2-EYFP were co-expressed in HEK293 cells, with each of the conserved hydrophobic residues in the αC-helix sequentially substituted to aspartic acid (Additional file 1: Figs. S8a, S9a). These substitutions were designed to disrupt the hydrophobicity of the putative helix-helix interface through the introduction of a negative charge. The median plasma membrane localization level of both POPDC isoforms across the cells was then determined as before and the difference to the wild-type pair analyzed. It was found that POPDC1 mutations F249D (0.812, 95% CI 0.584, 0.990; n = 56), I253D (0.841, 95% CI 0.571, 1.185; n = 27) and I257D (0.625, 95% CI 0.441, 0.741; n = 40) led to severe mislocalization of POPDC1 compared to the wild-type pair (3.676, 95% CI 3.176, 4.504; n = 34) (all *p* < 0.0001, Fig. 8e). These mutations had a similar effect on POPDC2 with F249D (0.845, 95% CI 0.650, 0.982; n = 56), I253D (1.202, 95% CI 0.790, 2.060; n = 27), and I257D (0.635, 95% CI 0.488, 0.779; n = 40) (all *p* < 0.0001) all leading to a reduction in the plasma membrane localization of POPDC2 compared to when co-expressed with wild-type POPDC1 (4.443, 95% CI 3.902, 6.662; n = 34). The other mutations within the αC-helix of POPDC1 did not lead to any significant changes in POPDC1 or POPDC2 plasma membrane localization (Fig. 8e). In POPDC2, the F233D (0.922, 95% CI 0.743, 1.277; n = 20), L237D (0.967, 95% CI 0.750, 1.267; n = 45), and I241D (0.735, 95% CI 0.642, 0.922; n = 42) mutations led to a significant disruption of POPDC1 plasma localization (all *p* < 0.0001) compared to when expressed with wild-type POPDC2 (Fig. 8f). These mutations also led to mislocalization of POPDC2 itself: F233D (0.778, 95% CI 0.651, 0.980; n = 20), L237D (1.121, 95% CI 0.860, 1.644; n = 45), and I241D (1.146, 95% CI 0.946, 1.479; n = 42) (all *p* < 0.0001). A mild reduction in POPDC2 plasma membrane localization was also seen with the I229D mutation (2.828, 95% CI 2.098, 3.337; n = 48, *p* = 0.049) (Fig. 8f). The L245D, L261D, and L264D mutations in POPDC1 and the I229D, L245D, and L248D in POPDC2, had no effect on the subcellular expression of either protein (except the minor change in POPDC2 expression in case of I229D). A loss in absolute plasma membrane expression was commonly seen in mutations that led to mislocalization of the proteins (Additional file 1: Figs. S8b, S9b), with lesser or no changes in cytoplasmic levels observed (Additional file 1: Figs. S8c, S9c). Residues that are aligned between POPDC1 and POPDC2 had very similar impacts on the plasma membrane localization of both isoforms (Fig. 8g, h). Mutation of the three residues at the core of the proposed αC-helices led to major losses in POPDC1 and POPDC2 plasma membrane localization, while substitution of the residues at the N- and C-termini of the helices had no, or only minimal effect (Fig. 8i).

## Discussion

The two probands homozygous for the *BVES* (c.547G > T, p.V183F) variant displayed a skeletal muscle-restricted phenotype, largely different from previous reports of *BVES* variants [5, 11, 40], especially since cardiological workup has not yet revealed signs of heart disease in either case. However, it remains possible that these patients might also develop a cardiac pathology later in life. The phenotypes in these patients likely represent a milder end of the clinical spectrum associated with this gene, starting in the posterior lower leg, progressing to the proximal lower limbs, with relative sparing of the upper body. Muscle pain with high CK and distal onset in the posterior lower leg has been described in patients with biallelic nonsense *BVES* variants [19]. The significant hyperCKemia associated with muscle necrosis on biopsy, onset as a calf myopathy with subsequent fast progression in adulthood, and the distribution of MRI changes (*adductor longus* and *magnus*, *gluteus minimus* and *semimembranosus*, together with abnormalities on T2-STIR images and some degree of asymmetry) closely resembled LGMDR12, which is associated with anoctamin 5 (*ANO5*) deficiency [32]. It is therefore noteworthy that POPDC1 has been shown to physically interact with anoctamin 5 via its cytoplasmic domain [29]. Notably, in both our patients extensive genetic testing did not reveal concomitant potentially pathogenic variants in other genes causative for myopathies, and a superimposed inflammatory myopathy in PT2 was not supported by muscle pathology, response to treatment or serological data.

In several previously reported cases of patients carrying a variant in *BVES* and developing LGMDR25, a loss in sarcolemmal localization of both POPDC1 and POPDC2 within skeletal muscle fibers was reported [11, 19, 40]. We therefore investigated the effect of the *BVES* p.V183F variant on the sarcolemmal expression of POPDC1 and POPDC2 in these patients. The mild reductions in the sarcolemmal expression level for both isoforms differed from the substantial loss of POPDC1 and POPDC2 at the sarcolemma in the patient carrying a homozygous *BVES* p.Q153X variant [15], as well as other previously reported *BVES* variants [11, 19, 40]. The homozygous *Popdc2*^*W188X/*W188X^ mouse, carrying a mutation recently identified in two families suffering from AV-block but with normal skeletal muscle function [37], showed an intermediate reduction in sarcolemmal expression of both isoforms. Our results demonstrate that mutations in either POPDC1 or POPDC2 may result in impaired membrane localization, suggesting that membrane trafficking is dependent on simultaneous expression of both proteins, but the severity of this effect is specific to each mutation. The difference in phenotype between patients carrying the p.V183F variant, and others such as the p.S201F missense variant, is probably determined by their differential effect on membrane localization, which was nearly normal in case of the p.V183F variant, while it was strongly affected in case of the p.S201F mutant. In addition, the morphology of the muscle fibers of patients possessing the POPDC1 p.V183F and the POPDC1 p.Q153X mutations were highly divergent. A fiber hypertrophy was observed in case of the biopsy taken from the patient carrying the POPDC1 p.Q153X variant, while fibers with a diverse range of cross-sectional areas, including a large population of hypotrophic fibers, was seen in both POPDC1 p.V183F patients, possibly reflecting an increased rate of degeneration/regeneration. In contrast only a very minor hypotrophy of muscle fibers was observed in the *Popdc2*^W188X/W188X^ mouse. It therefore seems that the different POPDC mutations lead to varying pathologies at the fiber level.

Previously, it has been reported that forced expression of POPDC1 in cell lines resulted mainly in intracellular expression, which contrasts with native skeletal and cardiac muscle where the protein is mainly localized at the sarcolemma [2, 18, 26, 40, 52]. Our finding that co-expressing POPDC1 and POPDC2 in HEK293 cells yielded a high degree of plasma membrane localization of both proteins supports the notion that co-expression of POPDC1 and POPDC2 is required for proper plasma membrane transport. It also permitted the use of HEK293 cells as a model system for investigating the effect of mutations on POPDC protein subcellular expression patterns. The disruption of plasma membrane expression and localization of POPDC1 and POPDC2 in HEK293 cells in the presence of POPDC1 p.Q153X, p.S201F, and POPDC2 p.W188X mutations, replicated their effects in skeletal muscle [40].

We explain the requirement for POPDC1 and POPDC2 to be both present for normal plasma membrane localization by the formation of heteromeric complexes, probably during translation or shortly thereafter. Direct POPDC1–POPDC2 interactions were shown to occur after forced expression as demonstrated by co-immunoprecipitation, qBRET and BiFC, with their presence confirmed in cardiac tissue by PLA and co-immunoprecipitation.

POPDC1 and POPDC2 show varying expression profiles across tissues and so are unlikely to always be present in stoichiometric amounts [7]. The qBRET and Western blot assays suggest that POPDC proteins may form both homo- and hetero-oligomers. While qBRET showed that POPDC1 and POPDC2 preferentially undergo heterodimer formation, the Western blot experiments suggested that higher order heteromers may also be able to form. An equilibrium between these homo- and heteromeric complexes may exist within cells in a tissue-specific manner. These assays do utilize non-native overexpression systems, as well as denaturing conditions in the case of the Western blot, which may lead to non-native oligomer formation. Further investigations into POPDC complex stoichiometries using native tissue would be beneficial.

The BiFC assay showed that the POPDC1 p.Q153X, p.S201F and POPDC2 p.W188X mutations impaired POPDC1–POPDC2 complex formation, while POPDC1 p.V183F had no effect. The correlation between subcellular expression changes and POPDC1–POPDC2 complex formation provides evidence that disruption of the POPDC1–POPDC2 interaction is likely responsible for the changes in subcellular localization in the muscle fibers of patients. We propose that the αC-helices of POPDC1 and POPDC2, found within the Popeye domain, form an heteromeric interface. The set of highly conserved hydrophobic residues, and the modelled structures of the αC-helix, means that such an interface would likely be pseudo-symmetrical supporting its function as an interaction domain [16]. Substitution of these hydrophobic residues with aspartic acid had specific and symmetrical effects on both proteins with the residues F249, I253, and I257 in POPDC1, and F233, L237, and I241 in POPDC2, which are aligned, shown to be particularly important for normal subcellular localization of both proteins in HEK293 cells. The loss of the αC-helix may be responsible for the changes in POPDC1–POPDC2 interactions, and subcellular expression, seen with the POPDC1 p.Q153X and POPDC2 p.W188X nonsense mutations. In contrast, the missense POPDC1 p.V183F variant is predicted to be positioned distal to the αC-helix, which may explain its relatively mild effect on POPDC protein interactions and subcellular expression.

The POPDC1 p.S201F mutation significantly altered the interaction and subcellular expression of POPDC1 and POPDC2, which has also been observed in patient tissue [40], despite being positioned outside the αC-helix. It is known that the S201F mutation reduces the cAMP binding affinity of POPDC1 by around 50% [40]. In CAP, cAMP binding leads to stabilization of the C-helix, which influences CAP dimerization [31, 34]. Modelling of cAMP binding to the Popeye domain suggests the αC-helix may be involved in cAMP binding, (Additional file 1: Fig. S10b). We also note that in the non-cAMP bound model of POPDC1, a H-bond network involving the hydroxyl group of S201 and the side chains of αC-helix residues K260 and D256 is predicted to form (Additional file 1: Fig. S10c). As such, cAMP binding may mediate the conformation of the αC-helix, and therefore POPDC1–POPDC2 interactions, possibly explaining the significant effect of the S201F mutation.

We also cannot exclude the possibility that the αC-helix mutations studied here led to a more generalized unfolding of the proteins, or changes in cAMP binding affinity, alongside, or instead of, specific effects on the POPDC1–POPDC2 interaction.

In all cases investigated here it is likely that POPDC1–POPDC2 interactions are not totally abolished, with above background BiFC signals seen in all cases. The preservation of co-immunoprecipitation of POPDC1 and POPDC2 when POPDC1 was subjected to a series of truncations, as well as with POPDC2 p.W188X, demonstrates that interaction may occur in the absence of the Popeye domain, via the N-terminal and/or transmembrane regions, although the exact nature of these possible interfaces is unknown (Additional file 1: Fig. S10a).

The BiFC assay utilizes split Venus tags at the cytoplasmic C-terminals of the POPDC isoforms. The reduction in BiFC signal in the presence of the POPDC1 p.Q153X, p.S201F and POPDC2 p.W188X mutations likely represents the effect of the disruption of the cytoplasmic Popeye domain-mediated interactions between the proteins. The N-terminal/transmembrane interface is less likely to be affected by the mutations within the Popeye domain, possibly explaining the preservation of co-immunoprecipitation and the presence of, albeit reduced, BiFC signal in the presence of these POPDC mutations. However, it appears that disruption of the Popeye domain-mediated interactions is sufficient to prevent normal POPDC1 and POPDC2 subcellular expression, even if other interfaces are intact.

While we still do not fully understand the mechanism underlying the parallel changes in the subcellular localization of POPDC1 and POPDC2 in response to single mutations, there is a strong precedent for the requirement of heteromeric interactions for correct trafficking of transmembrane proteins. Examples include the T cell antigen receptors [6] and the GABA_B_ receptors [9, 21, 22, 27, 55], which require complete heteromeric interactions between the subunits of the receptor complex to avoid endoplasmic reticulum (ER) retention and subsequent degradation. Several possible ER retention motifs are present within the Popeye domain and C-terminal domain of POPDC1 and POPDC2. Heteromeric Popeye domain interactions through the αC-helices may be required to mask ER retention motifs in POPDC1 and POPDC2 and permit correct movement of the complex to the plasma membrane.

Given that the POPDC1 p.V183F mutation only mildly alters POPDC1 and POPDC2 subcellular expression in skeletal muscle, with no effect in HEK293 cells, the reason for its pathogenicity is less clear. One hypothesis is that the V183F mutation may potentially affect a binding site for an unknown interaction partner, which is essential for skeletal muscle but not present, or redundant, in the heart. Recently, a binding site for PDE4 was mapped to the β3-strand of POPDC1 [47], adjacent to the β4-strand location of V183. By analogy, we hypothesize that an additional interaction site is altered, in an allosteric manner given the internal orientation of the V183 sidechain, as a result of the V183F mutation. However, the full effect of this mutation on POPDC1 function is yet to be determined.

In native tissue, POPDC1–POPDC2 interactions are unlikely to be the only factor controlling POPDC protein expression and localization. Homomeric complex formation of POPDC1 was first described in the chicken heart when tissue extracts were subjected to Western blot analysis [52]. Under non-reducing conditions an immunoreactive band was observed, which was double the size of the band seen under reducing conditions. Moreover, homomeric POPDC1 interactions have previously been suggested to be involved in mediating subcellular localization [38]. Interestingly, the homomeric interaction of POPDC1 was mapped to sequences immediately C-terminal to the αC-helix [23, 26], suggesting that this interface may also support homomeric interactions. We also identified an interaction between POPDC1 and POPDC3, although POPDC2 and POPDC3 do not appear to form direct complexes. Although we have not further investigated whether POPDC1 and POPDC3 expression would also support membrane localization the high level of isoform homology, particularly at the αC-helix, suggests this may be possible.

*BVES* and *POPDC3* mutations display recessive inheritance, while *POPDC2* mutations show a dominant mode [5, 10, 11, 15, 17, 19, 37, 40, 50, 53, 57]. If disruption of stoichiometric POPDC1–POPDC2 complexes played an equal role in the mechanism of mutations of both of isoforms, identical modes of inheritance would be expected. As mentioned, POPDC isoforms are differentially expressed across tissues, and this is also reflected in the observed phenotypes of patients expressing variants of these proteins. Patients carrying *POPDC2* variants show only cardiac arrhythmias [17, 37], while *POPDC3* variants are only associated with LGMD [50, 53, 57]. In contrast, patients carrying *BVES* variants develop cardiac and/or skeletal muscle phenotypes [5, 10, 11, 15, 19, 40]. Given that all three POPDC isoforms are expressed in skeletal muscle, it is possible that a loss of POPDC2 could be compensated by POPDC3. The weak expression of *POPDC3* in cardiac muscle may be responsible for the exclusive cardiac phenotype in patients carrying *POPDC2* variants. The near equal expression of *BVES* in both skeletal and cardiac muscle likely explains the cardiac and/or skeletal muscle phenotypes seen in patients carrying mutations in *BVES*.

As well as mutations leading to changes in POPDC protein interfaces, the loss of mutant protein expression must also be considered. A reduction of POPDC1 protein levels in the patient possessing the *BVES* (c.457C > T, p.Q153X) mutation was previously suggested to be due to nonsense mediated decay (NMD) [15], although Rinné et al. detected *POPDC2* (c.563G > A, p.W188X) mutant transcript and protein in patient’s leukocytes, suggesting that NMD did not totally prevent expression of the mutant protein in that case [37].

POPDC1 and POPDC2 have been reported to interact with a number of proteins linked to sarcolemmal stability or repair such as dystrophin [40], dysferlin [40], Xin-related protein 1 [18], annexin A5 [18], anoctamin 5 [29], and caveolin-3 [1]. Given the presence of POPDC proteins in numerous complexes, it is likely that changes in POPDC protein expression has wide-ranging effects, which may contribute to the observed muscular pathologies and hyperCKemia observed in patients. However, while TREK-1 expression seems to be dependent on POPDC1 [40], it has been reported that the POPDC1 p.S201F mutation had no effect on dysferlin, dystrophin and caveolin-3 expression [40]. Whether these additional interaction partners also influence POPDC protein localization has not yet been determined. While POPDC1–POPDC2 interactions are clearly highly important, it is likely that a combination of homomeric and heteromeric POPDC interactions, together with complexation with other proteins, are responsible for the full control of POPDC protein subcellular expression in vivo.

The subcellular expression POPDC1–POPDC2 complexes, may influence the assembly of cAMP signaling complexes and nanodomains, which are vital in cAMP dependent signaling [3, 56]. Indeed, POPDC1 has been shown to form functionally important interactions with cAMP pathway proteins such as PDE4, AC9, and TREK-1 in the heart, the disruption of which may impact calcium transients, β-adrenergic signaling, and ion channel function [4, 47]. The impact of POPDC protein mislocalisation on cAMP signalosomes could explain the cardiac arrhythmias which have been observed in many patients carrying *BVES* or *POPDC2* variants [5, 10, 11, 15, 19, 37, 40] and may also be responsible for the muscular dystrophy phenotype associated with POPDC mutations, although the link to cAMP signaling has not yet been established in the case of skeletal muscle.

## Conclusions

We describe a novel variant in *BVES* (c.547G > T, p.V183F), which in contrast to other *BVES* variants retains nearly normal membrane trafficking of both POPDC1 and POPDC2. We demonstrate that membrane targeting is largely determined by the heteromeric interaction of POPDC proteins via an array of evolutionary conserved hydrophobic interface residues located in the αC-helix of the Popeye domain. Given the relevance of POPDC1–POPDC2 interactions for sarcolemmal expression, and impaired membrane localization in patients carrying POPDC mutations, we recommend establishing whether novel POPDC mutations alter POPDC protein–protein interactions and assessing membrane localization in tissue biopsies. Where biopsies are not available, a co-transfection analysis in HEK293 cells appears to be suitable as a surrogate. Unravelling the effects of a loss of POPDC1–POPDC2 complexes at the sarcolemma and its impact on the array of POPDC-interacting proteins known to be required for normal skeletal muscle and cardiomyocyte function, and/or cAMP signaling, will be required to understand the range of phenotypes seen in patients carrying POPDC gene mutations.

## Supplementary Information

**Additional file 1: Fig. S1**. Expression analysis of POPDC1 and POPDC2 in skeletal muscle biopsies of patients carrying a *POPDC1* p.V183F mutation. **a**–**c** POPDC1 and **d**–**f** POPDC2 expression in **a** and **d** sarcolemma, **b** and **e** cytoplasm and **c** and **f** the ratio of sarcolemma/cytoplasm expression as determined by immunostaining. **g** Quantitative comparison of the median cross-sectional area of muscle fibers in patient and control samples. In **a**, **b**, **d** and **e** the median level in each control was set to 1. Dashed lines indicate the normalised median and interquartile range. Data were analysed using a Mann–Whitney test. ****p < 0.0001. **Fig. S2**. Expression analysis of POPDC1 and POPDC2 in skeletal muscle biopsy of a patient carrying a *POPDC1* p.Q153X mutation. Expression of **a**–**c** POPDC1 and **d**–**f** POPDC2 in **a** and **d** the sarcolemma, **b** and **e** cytoplasm and **c** and **f** the ratio of sarcolemma/cytoplasm expression as determined by immunostaining. **g** Quantitative comparison of the median cross-sectional area of muscle fibers in patient and control samples. In **a**, **b**, **d** and **e** the median level in the control was set to one. Dashed lines indicate the normalised median and interquartile range. Data were analysed using Mann–Whitney test. *p < 0.05, ****p < 0.0001. **Fig. S3**. Expression analysis of POPDC1 and POPDC2 in skeletal muscle of homozygous mice carrying a *Popdc2* p.W188X mutation and a WT control. Expression analysis of **a**–**c** POPDC1 and **d**–**f** POPDC2 in **a** and **d** the sarcolemma, **b** and **e** cytoplasm and **c** and **f** the ratio of sarcolemma/cytoplasm expression as determined by immunostaining. **g** Quantitative comparison of the median crosssectional area of muscle fibers in mutant and control samples. In **a**, **b**, **d** and **e** the median level in the control was set to one. Dashed lines indicate the normalised median and interquartile range. Data were analysed using a Mann–Whitney test. *p < 0.05, ***p < 0.001, ****p < 0.0001. **Fig. S4**. Expression of POPDC1 and POPDC2 in HEK293 cells after transfection with different POPDC1 and POPDC2 constructs. **a** Normalized absolute expression levels of POPDC1-ECFP and POPDC2-EYFP in the cytoplasm and **b** plasma membrane when co-expressed or expressed alone in HEK293 cells. Bars show median ± 95% CI. Single (POPDC1-ECFP n = 9, POPDC2-ECFP n = 9) or co-expression conditions (n = 46) were compared using a Mann–Whitney test. **c** Normalized absolute expression levels of POPDC1-ECFP and POPDC2-EYFP in the cytoplasm and **d** plasma membrane when co-expressed with wildtype (n = 46) or mutant (POPDC1 p.V183F n = 47, POPDC1 p.Q153X n = 17, POPDC1 p.S201F n = 22, POPDC2 p.W188X n = 24) POPDC dimerization partner in HEK293 cells. Bars show median ± 95% CI. Groups were compared using Kruskal–Wallis followed by Dunn’s test using the wildtype pair for comparison; *p < 0.05, ****p < 0.0001. **Fig. S5**. Total BRET expression of POPDC isoforms. **a**–**c** The total expression level of POPDC-NL and POPDC-HT constructs in HEK293 cells when expressed at different ratios of **a** POPDC1-NL + POPDC1-HT, **b** POPDC2-NL + POPDC2-HT, **c** POPDC1-HT + POPDC2-NL. The plots were fitted to a horizontal line and compared to a nonhorizontal fit using an F-test with the results shown as insets. Only ratios of POPDCHT: POPDC-NL expression levels above two were included, as recommended [1]. *p* > 0.05 indicates acceptance of a horizontal fit with no significant difference in total POPDC expression at different expression ratios. **Fig. S6**. Analysis of bimolecular fluorescence complementation (BiFC) in transfected HEK293 cells. Confocal microscopy of HEK293 cells after co-transfection of POPDC1-VC or POPDC2-VN with either POPDC1 p.V183F-VC, POPDC1 p.Q153XVC, POPDC1 p.S201F-VC, POPDC2 p.W188X-VN, respectively. Insets show the boxed area at higher magnification. mRFP was used as internal expression control. The BiFC signal (Venus fluorescence) normalized to mRFP expression was measured only from cells expressing significant levels of mRFP. Nuclei were stained with Hoechst-33342. 5–9 images were taken per group over a minimum of 2 transfections for each construct. Scale bar: 200 μm. **Fig. S7**. Structural models of CAP protein from E. coli. **a** Structure of a CAP dimer in the cAMP bound form (PDB: 1G6N) [2]. **b** The C-helix interface that modulates CAP dimerization. **c** Interaction of residues of the C-helices of CAP with cAMP. **Fig. S8**. Membrane trafficking after co-transfection of αC-helix mutants of POPDC1 and wildtype POPDC2. **a** POPDC2-EYFP was expressed in HEK293 cells with wildtype POPDC1-ECFP and a series of constructs in which one of the conserved hydrophobic residues within the αC-helix had been mutated to an aspartic acid. Scale bar: 10 μm. **b** and **c** Quantification of the normalised expression levels of POPDC1-ECFP and POPDC2-EYFP in the **b** plasma membrane and **c** cytoplasm as a function of the different POPDC1 mutations. Total number of cells analyzed: WT n = 34, L245D n = 71, F249D n = 56, I253D n = 27, I257D n = 40, L261D n = 47, L264D n = 42. Min two transfections per group. Bars show median ± 95% CI. Groups were compared using Kruskal–Wallis followed by Dunn’s test using the wildtype pair as a comparison; **p < 0.01, ****p < 0.0001. **Fig. S9**. Membrane trafficking after co-transfection of wildtype POPDC1 and αC-helix mutants of POPDC2. **a** POPDC1-ECFP was expressed in HEK293 cells together with wildtype POPDC2-EYFP or a construct in which one of the conserved hydrophobic residues within the αC-helix of POPDC2 had been mutated to an aspartic acid. The plasma membrane was marked using DiD. Scale bar: 10 μm. **b** and **c** Quantification of the normalized expression levels of POPDC1-ECFP and POPDC2-EYFP in the **b** plasma membrane and **c** cytoplasm as a function of the different POPDC2 mutations. Total number of cells analyzed: WT n = 34, I229D n = 48, F233D n = 20, L237D n = 45, I241D n = 42, L245D n = 63, L248D n = 56. Min 2 transfections per group. Bars show median ± 95% CI. Groups were compared using Kruskal–Wallis followed by Dunn’s test using the wildtype pair as a comparison; *p < 0.05, ***p < 0.005, ****p < 0.0001. **Fig. S10**. The role of the αC-helix in the function of POPDC proteins. **a** Model of the protein domains mediating the interaction of POPDC1 and POPDC2. The interaction between POPDC1 and POPDC2 is proposed to be mediated through an interface between the αC-helices (red). Another interaction via the N-terminus and/or transmembrane domains (grey) is also depicted. **b** The predicted binding mode of cAMP to the Popeye domain of POPDC1, as determined by the 3DLigandSite server [3]. The side chains of K260 and L264 are shown. **c** The side chains of the ultra-conserved S201,D256 and K260, in POPDC1 are predicted to form a H-bond network connecting the PBC of the Popeye domain with the αC-helix. Additional H-bonds formed by atoms from the peptide bond are also depicted. **Table S1**. Primer sequences used in site-directed mutagenesis of POPDC1-ECFP or POPDC2-EYFP.

## Data Availability

Data sharing is not applicable to this article as no datasets were generated or analysed during the current study.

## Abbreviations

AC9: Adenylyl cyclase 9
ANO5: Anoctamin 5
AV: Atrioventricular
BiFC: Bimolecular fluorescence complementation
BRET: Bioluminescence resonance energy transfer
BVES: Blood vessel epicardial substance
cAMP: 3′,5′-Cyclic adenosine monophosphate
CCS: Cardiac conduction system
CI: Confidence interval
CK: Creatine kinase
CNBD: Cyclic nucleotide binding domain
CT1/2: Control 1/2
DAPI: 4′, 6-Diamidino-2-phenylindole dihydrochloride
DiD: 1,1′-Dioctadecyl-3,3,3′,3′-tetramethylindo-dicarbocyanine
DMSO: Dimethyl sulfoxide
ECFP: Enhanced cyan fluorescent protein
EYFP: Enhanced yellow fluorescent protein
fib: Fibers
img: Images
KI: Knockin
KO: Knockout
LGMD: Limb-girdle muscular dystrophy
MRI: Magnet resonance imaging
NMD: Nonsense mediated decay
PDE4: Phosphodiesterase 4
PBC: Phosphate binding cassette
PLA: Proximity ligation analysis
POPDC: Popeye domain containing
PT1/2: Patient 1/2
sec: Sections
SGCA: α-Sarcoglycan
VUS: Variant of unknown significance
