# Correction: Differential effects of mutations of POPDC proteins on heteromeric interaction and membrane trafficking

**DOI:** 10.1186/s40478-023-01591-0

**Published:** 2023-07-11

**Authors:** Alexander H. Swan, Roland F. R. Schindler, Marco Savarese, Isabelle Mayer, Susanne Rinné, Felix Bleser, Anne Schänzer, Andreas Hahn, Mario Sabatelli, Francesco Perna, Kathryn Chapman, Mark Pfuhl, Alan C. Spivey, Niels Decher, Bjarne Udd, Giorgio Tasca, Thomas Brand

**Affiliations:** 1 National Heart and Lung Institute (NHLI), Imperial College London, London, UK; 2 Department of Chemistry, Imperial College London, London, UK; 3 Department of Medical Genetics, Medicum, University of Helsinki, Helsinki, Finland; 4 Institute for Physiology and Pathophysiology, Vegetative Physiology, Philipps-University of Marburg, Marburg, Germany; 5 Institute of Neuropathology, Justus Liebig University Giessen, Giessen, Germany; 6 Department of Child Neurology, Justus Liebig University Giessen, Giessen, Germany; 7 Department of Neurology, Universitá Cattolica del Sacro Cuore, Rome, Italy; 8 Dipartimento Di Scienze Cardiovascolari, Fondazione Policlinico Universitario A. Gemelli IRCCS, Rome, Italy; 9 Assay Biology, Domainex Ltd, Cambridge, CB10 1XL UK; 10 School of Cardiovascular Medicine and Sciences and Randall Centre, King’s College London, London, UK; 11 Folkhälsan Research Center, University of Helsinki, Helsinki, Finland; 12 Unità Operativa Complessa di Neurologia, Fondazione Policlinico Universitario A. Gemelli IRCCS, Rome, Italy; 13 Present Address: John Walton Muscular Dystrophy Research Centre, Newcastle University and Newcastle Hospitals NHS Foundation Trusts , Newcastle Upon Tyne, UK; 14 Imperial Centre of Translational and Experimental Medicine, Du Cane Road, London, W120NN UK

**Correction: Acta Neuropathologica Communications (2023) 11:4** 10.1186/s40478-022-01501-w

Following publication of the original article [1], the authors identified errors in the affiliations assignment and in Fig. 3.

The affiliation of the author Niels Decher was incorrect. The correct affiliation is affiliation 4 (Institute for Physiology and Pathophysiology, Vegetative Physiology, Philipps-University of Marburg, Marburg, Germany), instead of affiliation 5 (Institute of Neuropathology, Justus Liebig University Giessen, Giessen, Germany).

For Fig. 3, the authors reported that a duplicated version of Fig. 2 was published as Fig. 3.

**Fig. 3:**
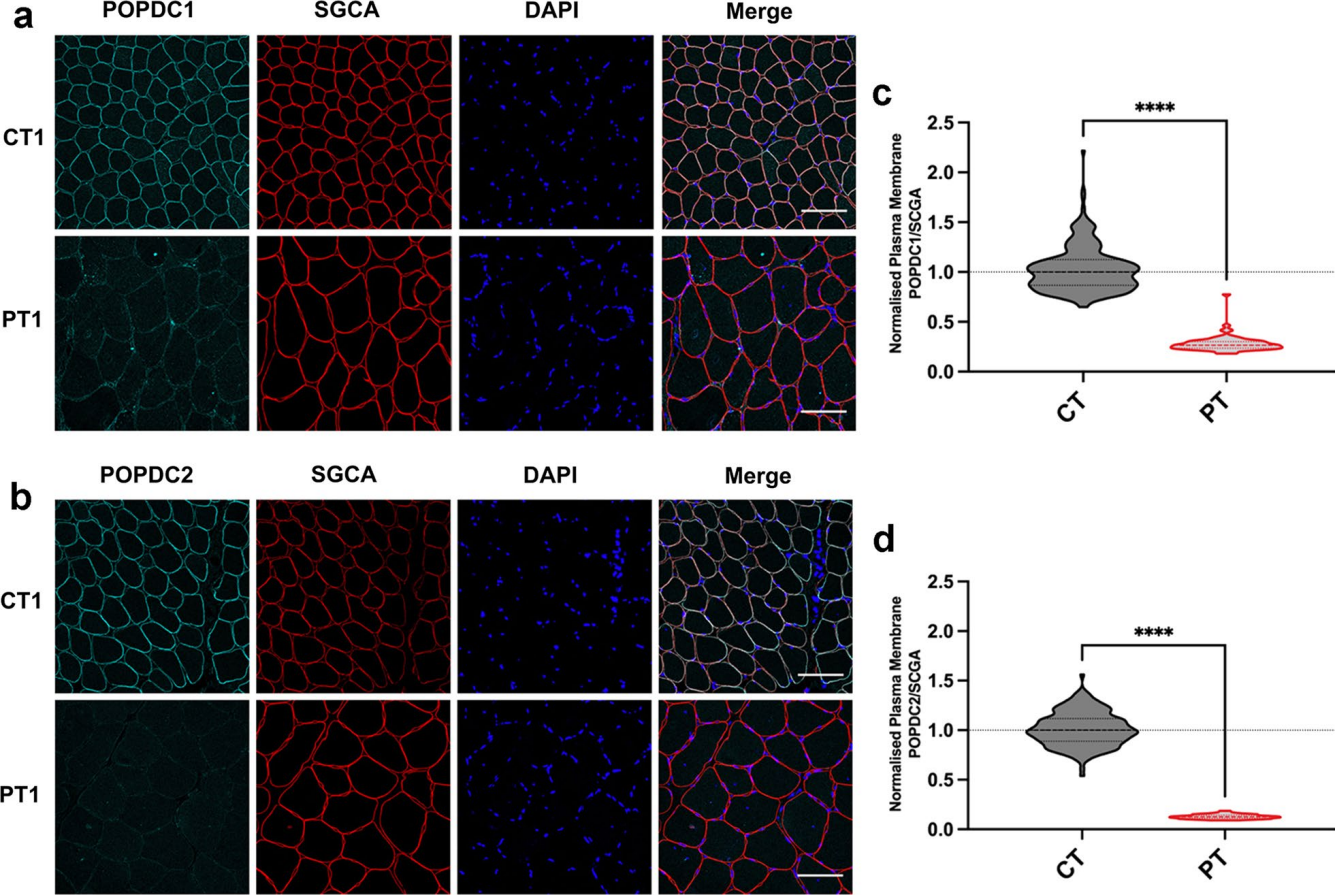
The expression of POPDC1 and POPDC2 is greatly reduced at the sarcolemma of skeletal muscle fibers expressing *POPDC1* p.Q153X. **a** and **b** Transverse sections of skeletal muscle biopsies from a patient (PT) carrying the *POPDC1* p.Q153X variant in homozygosity and a matched control (CT) were stained for **a** POPDC1 or **b** POPDC2, along with SGCA as a sarcolemma marker. Scale bar: 100 μm. **c** and **d** The expression levels of **c** POPDC1 and **d** POPDC2 in the sarcolemma normalized to SGCA, were quantified in individual fibers. The number of sections (sec), images (img) and fibers (fib) analyzed per group are as follows: CT: POPDC1—1 s, 4 img, 238 fib; POPDC2—1 s, 4 img, 163 fib. PT: POPDC1—1 s, 3 img, 65 fib; POPDC2—1 s, 3 img, 70 fib. The median POPDC/SGCA-level in each control biopsy was set to 1. Dashed lines indicate the normalized median and interquartile range. Data were analyzed using Mann–Whitney test; ****p < 0.0001

The corrected affiliation assignment and Fig. 3 have been provided in this Correction article and the original article [1] has been updated
