# Supplementary material for: Differential effects of mutations of POPDC proteins on heteromeric interaction and membrane trafficking
Source: Acta Neuropathol Commun. 2023 Jan 9;11:4. doi: 10.1186/s40478-022-01501-w (PMC9830914; doi:10.1186/s40478-022-01501-w)
Supplement: Supplementary file 1 — Additional file 1: Fig. S1. Expression analysis of POPDC1 and POPDC2 in skeletal muscle biopsies of patients carrying a POPDC1 p.V183F mutation. a–c POPDC1 and d–f POPDC2 expression in a and d sarcolemma, b and e cytoplasm and c and f the ratio of sarcolemma/cytoplasm expression as determined by immunostaining. g Quantitative comparison of the median cross-sectional area of muscle fibers in patient and control samples. In a, b, d and e the median level in each control was set to 1. Dashed lines indicate the normalised median and interquartile range. Data were analysed using a Mann–Whitney test. ****p < 0.0001. Fig. S2. Expression analysis of POPDC1 and POPDC2 in skeletal muscle biopsy of a patient carrying a POPDC1 p.Q153X mutation. Expression of a–c POPDC1 and d–f POPDC2 in a and d the sarcolemma, b and e cytoplasm and c and f the ratio of sarcolemma/cytoplasm expression as determined by immunostaining. g Quantitative comparison of the median cross-sectional area of muscle fibers in patient and control samples. In a, b, d and e the median level in the control was set to one. Dashed lines indicate the normalised median and interquartile range. Data were analysed using Mann–Whitney test. *p < 0.05, ****p < 0.0001. Fig. S3. Expression analysis of POPDC1 and POPDC2 in skeletal muscle of homozygous mice carrying a Popdc2 p.W188X mutation and a WT control. Expression analysis of a–c POPDC1 and d–f POPDC2 in a and d the sarcolemma, b and e cytoplasm and c and f the ratio of sarcolemma/cytoplasm expression as determined by immunostaining. g Quantitative comparison of the median crosssectional area of muscle fibers in mutant and control samples. In a, b, d and e the median level in the control was set to one. Dashed lines indicate the normalised median and interquartile range. Data were analysed using a Mann–Whitney test. *p < 0.05, ***p < 0.001, ****p < 0.0001. Fig. S4. Expression of POPDC1 and POPDC2 in HEK293 cells after transfection with different POPDC1 and POPDC2 cons [file 40478_2022_1501_MOESM1_ESM.pdf]

## Additional File 1

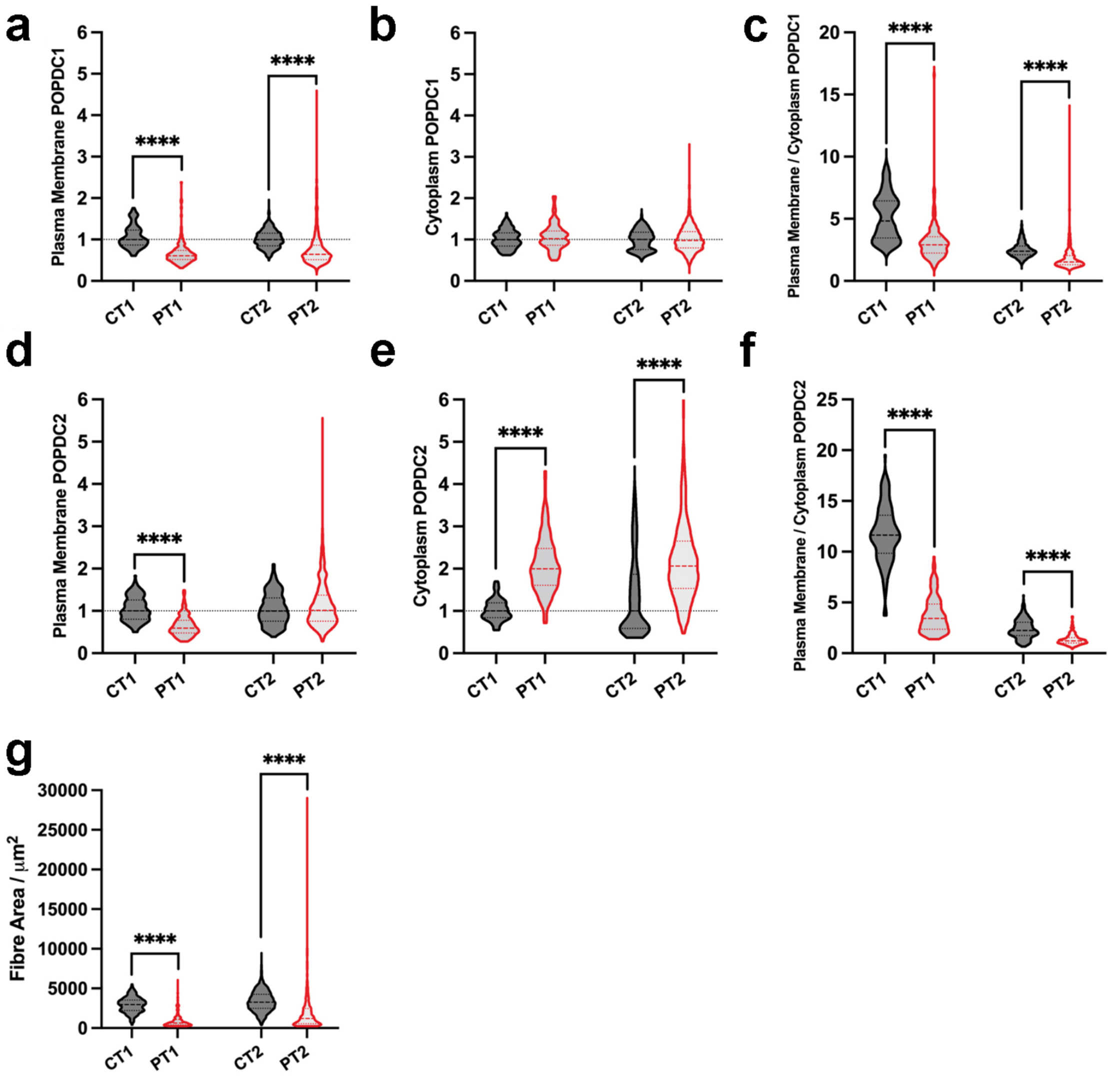


**Fig. S1** Expression analysis of POPDC1 and POPDC2 in skeletal muscle biopsies of patients carrying a *POPDC1* p.V183F mutation. **a**-**c** POPDC1 and **d**-**f** POPDC2 expression in **a** and **d** sarcolemma, **b** and **e** cytoplasm and **c** and **f** the ratio of sarcolemma/cytoplasm expression as determined by immunostaining. **g** Quantitative comparison of the median cross-sectional area of muscle fibers in patient and control samples. In **a**, **b**, **d** and **e** the median level in each control was set to 1. Dashed lines indicate the normalised median and interquartile range. Data were analysed using a Mann-Whitney test. **** *p* <0.0001.

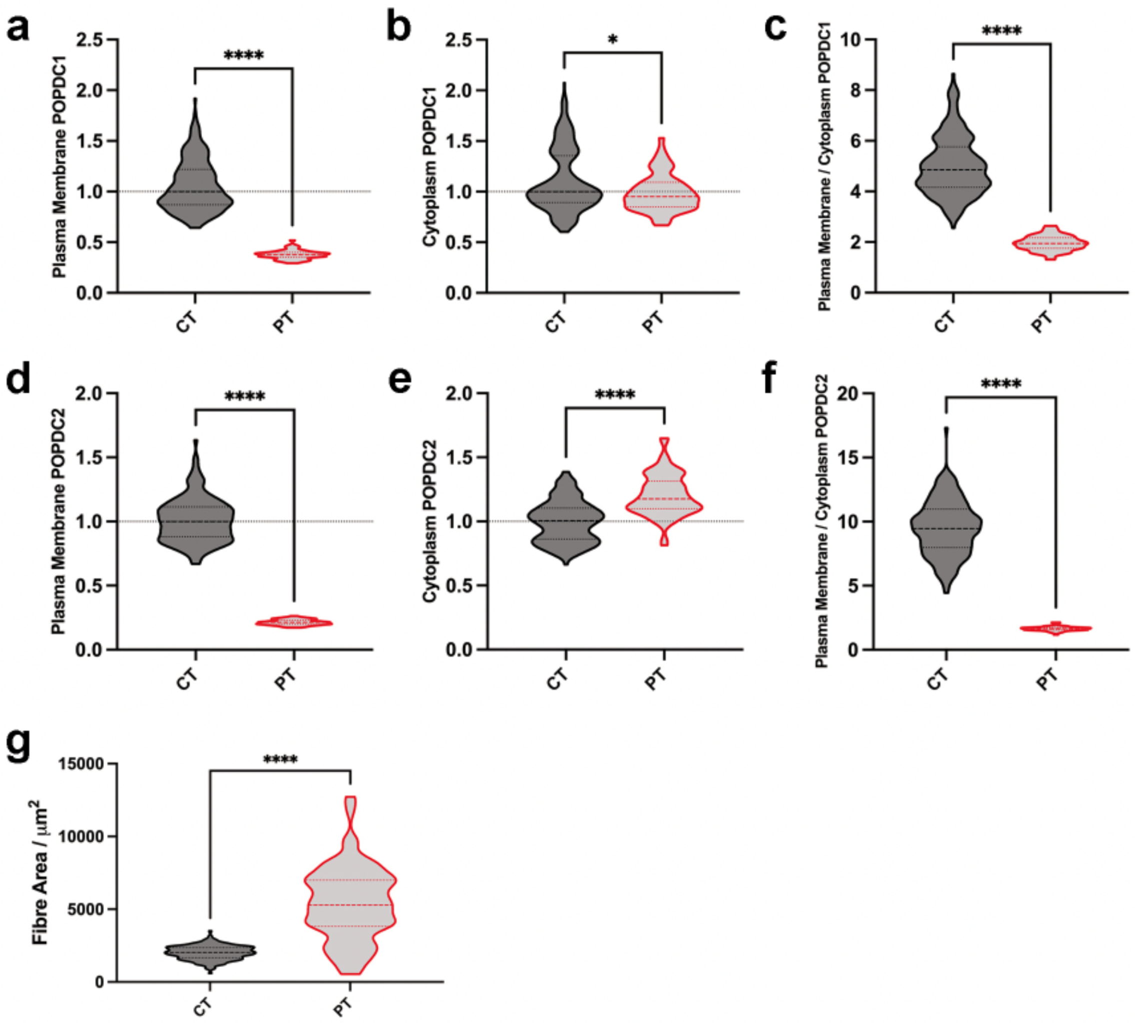


**Fig. S2** Expression analysis of POPDC1 and POPDC2 in skeletal muscle biopsy of a patient carrying a *POPDC1* p.Q153X mutation. Expression of **a**-**c** POPDC1 and **d**-**f** POPDC2 in **a** and **d** the sarcolemma, **b** and **e** cytoplasm and **c** and **f** the ratio of sarcolemma/cytoplasm expression as determined by immunostaining. **g** Quantitative comparison of the median cross-sectional area of muscle fibers in patient and control samples. In **a**, **b**, **d** and **e** the median level in the control was set to one. Dashed lines indicate the normalised median and interquartile range. Data were analysed using Mann-Whitney test. * $p < 0.05$, **** $p < 0.0001$.

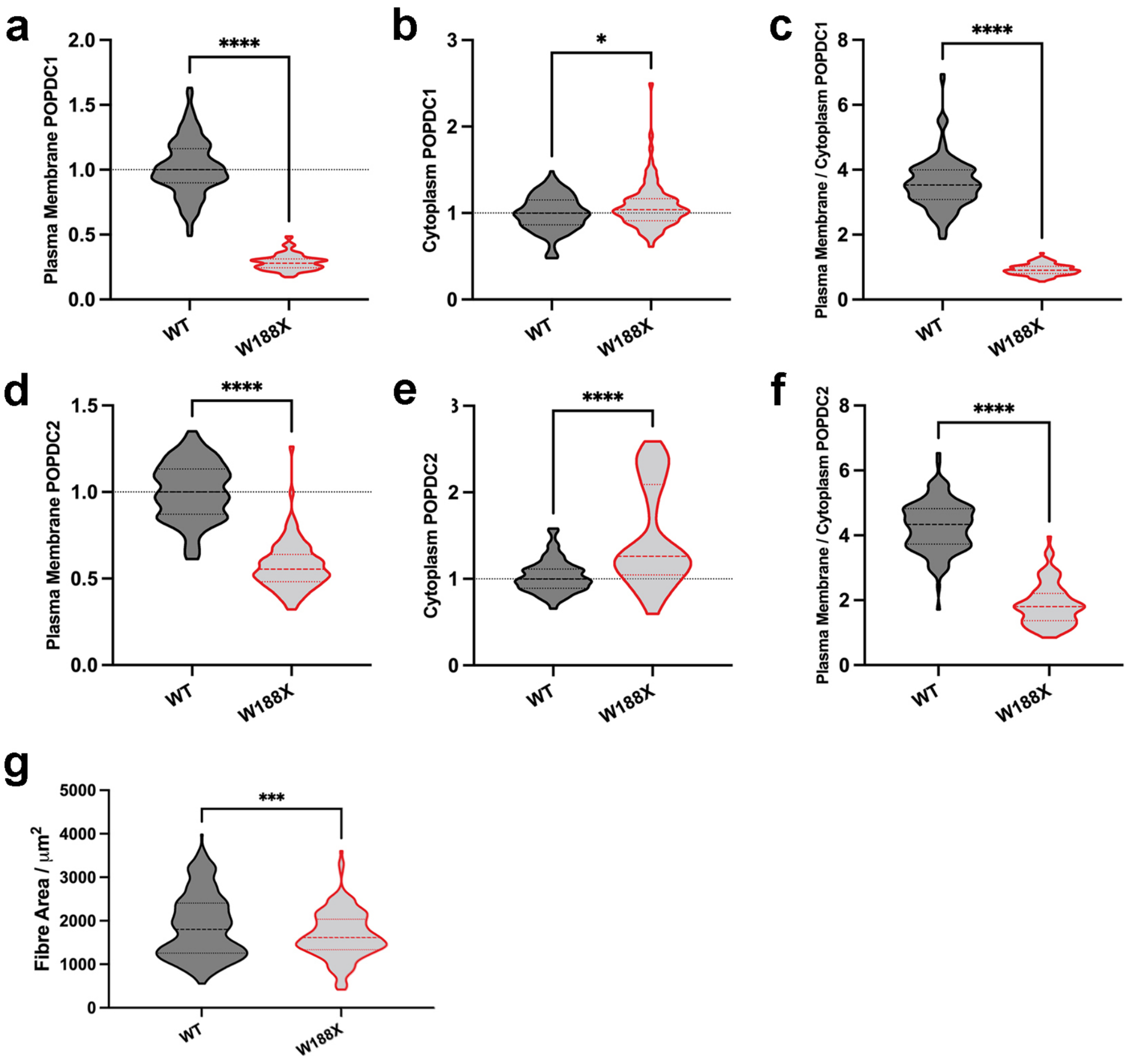


**Fig. S3** Expression analysis of POPDC1 and POPDC2 in skeletal muscle of homozygous mice carrying a *Popdc2* p.W188X mutation and a WT control. Expression analysis of **a**-**c** POPDC1 and **d**-**f** POPDC2 in **a** and **d** the sarcolemma, **b** and **e** cytoplasm and **c** and **f** the ratio of sarcolemma/cytoplasm expression as determined by immunostaining. **g** Quantitative comparison of the median cross-sectional area of muscle fibers in mutant and control samples. In **a**, **b**, **d** and **e** the median level in the control was set to one. Dashed lines indicate the normalised median and interquartile range. Data were analysed using a Mann-Whitney test. * $p<0.05$, *** $p<0.001$, **** $p<0.0001$.

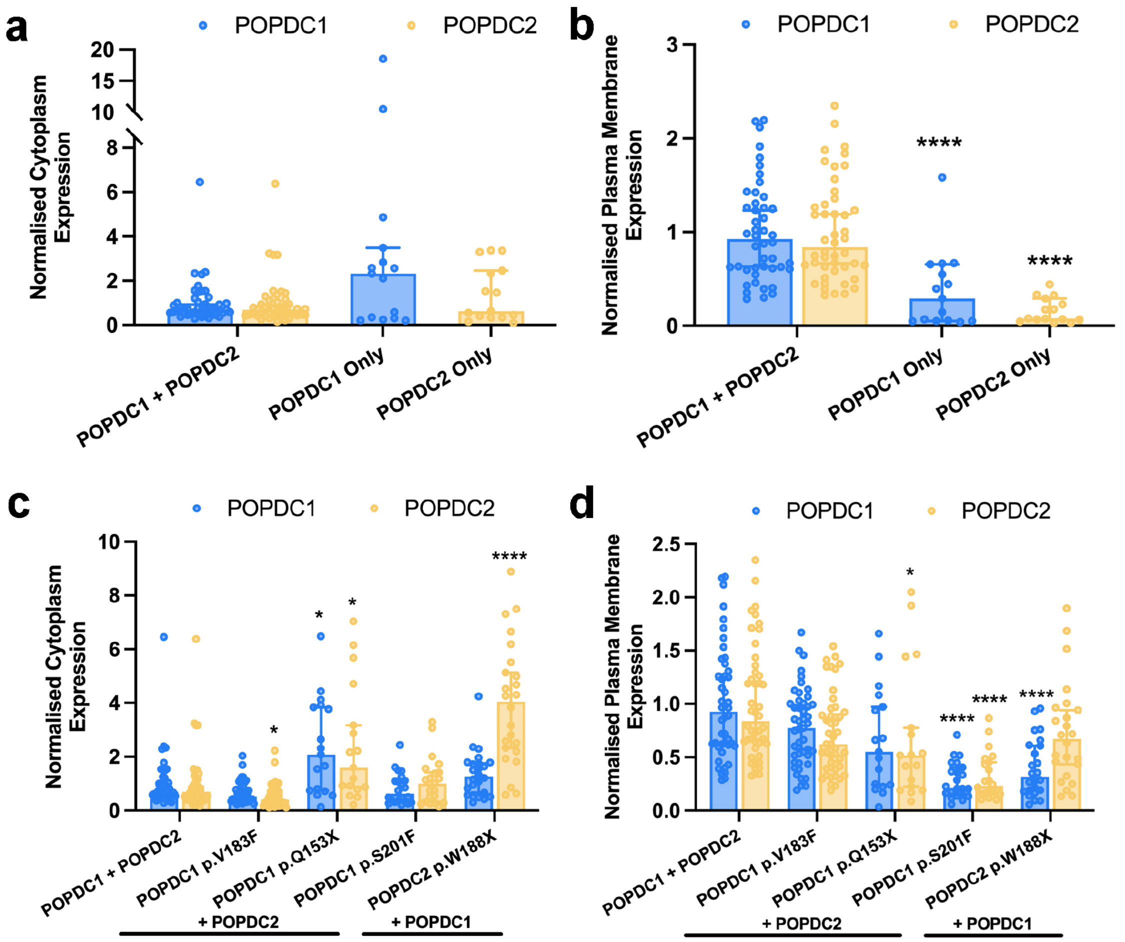


**Fig. S4** Expression of POPDC1 and POPDC2 in HEK293 cells after transfection with different POPDC1 and POPDC2 constructs. **a** Normalized absolute expression levels of POPDC1-ECFP and POPDC2-EYFP in the cytoplasm and **b** plasma membrane when co-expressed or expressed alone in HEK293 cells. Bars show median ± 95% CI. Single (POPDC1-ECFP n=9, POPDC2-ECFP n=9) or co-expression conditions (n=46) were compared using a Mann-Whitney test. **c** Normalized absolute expression levels of POPDC1-ECFP and POPDC2-EYFP in the cytoplasm and **d** plasma membrane when co-expressed with wildtype (n=46) or mutant (POPDC1 p.V183F n=47, POPDC1 p.Q153X n=17, POPDC1 p.S201F n=22, POPDC2 p.W188X n=24) POPDC dimerization partner in HEK293 cells. Bars show median ± 95% CI. Groups were compared using Kruskal-Wallis followed by Dunn's test using the wildtype pair for comparison; * *p* <0.05, **** *p* <0.0001.

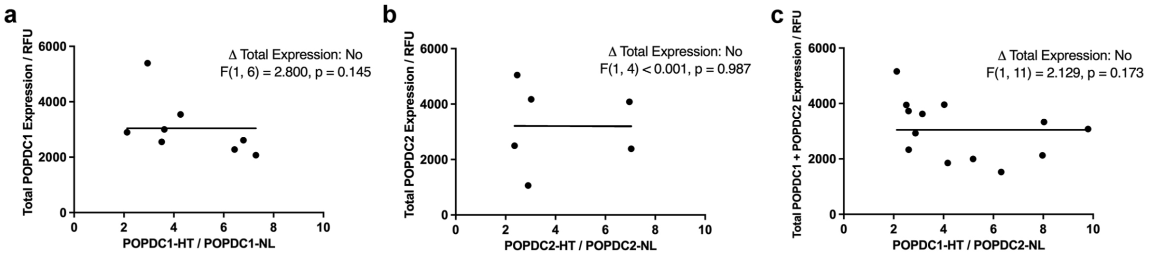


**Fig. S5** Total BRET expression of POPDC isoforms. **a**-**c** The total expression level of POPDC-NL and POPDC-HT constructs in HEK293 cells when expressed at different ratios of **a** POPDC1-NL + POPDC1-HT, **b** POPDC2-NL + POPDC2-HT, **c** POPDC1-HT + POPDC2-NL. The plots were fitted to a horizontal line and compared to a non-horizontal fit using an F-test with the results shown as insets. Only ratios of POPDC-HT:POPDC-NL expression levels above two were included, as recommended [1]. $p > 0.05$ indicates acceptance of a horizontal fit with no significant difference in total POPDC expression at different expression ratios.

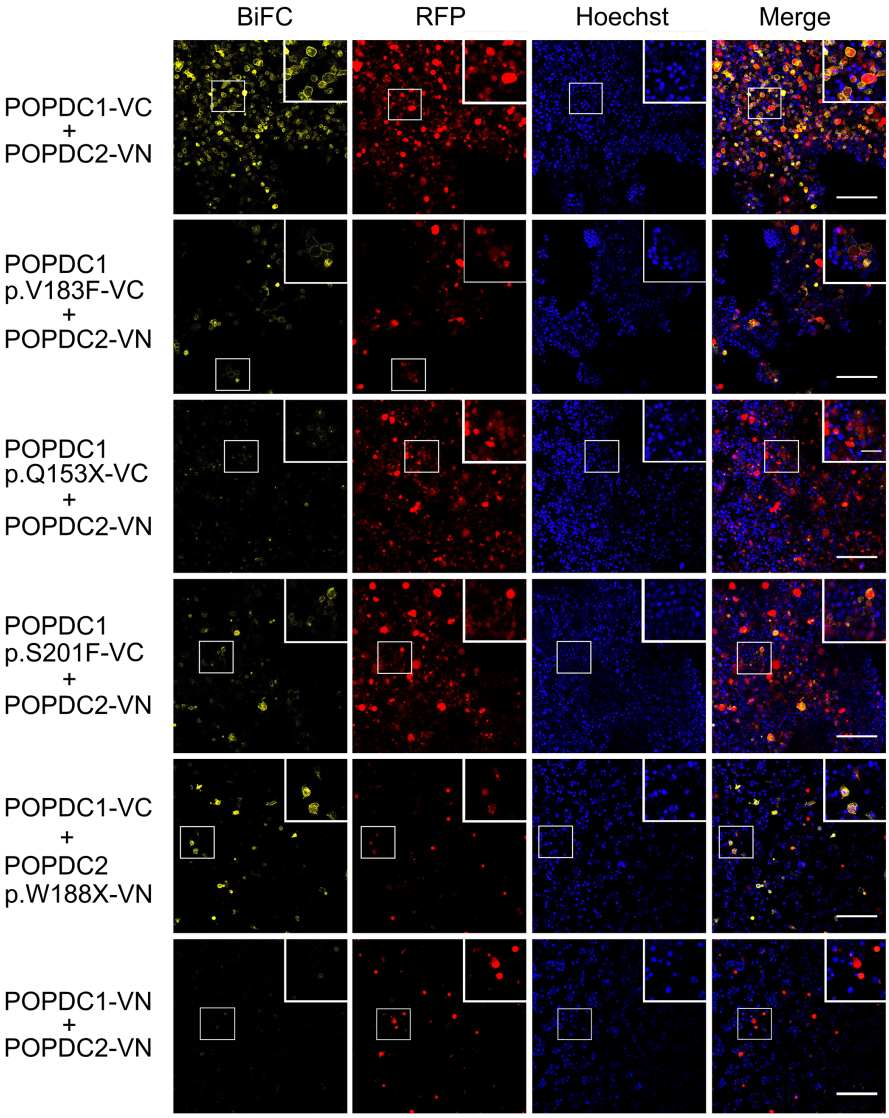


**Fig. S6** Analysis of bimolecular fluorescence complementation (BiFC) in transfected HEK293 cells. Confocal microscopy of HEK293 cells after co-transfection of POPDC1-VC or POPDC2-VN with either POPDC1 p.V183F-VC, POPDC1 p.Q153X-VC, POPDC1 p.S201F-VC, POPDC2 p.W188X-VN, respectively. Insets show the boxed area at higher magnification. mRFP was used as internal expression control. The BiFC signal (Venus fluorescence) normalized to mRFP expression was measured only from cells expressing significant levels of mRFP. Nuclei were stained with Hoechst-33342. 5-9 images were taken per group over a minimum of 2 transfections for each construct. Scale bar: 200 µm.

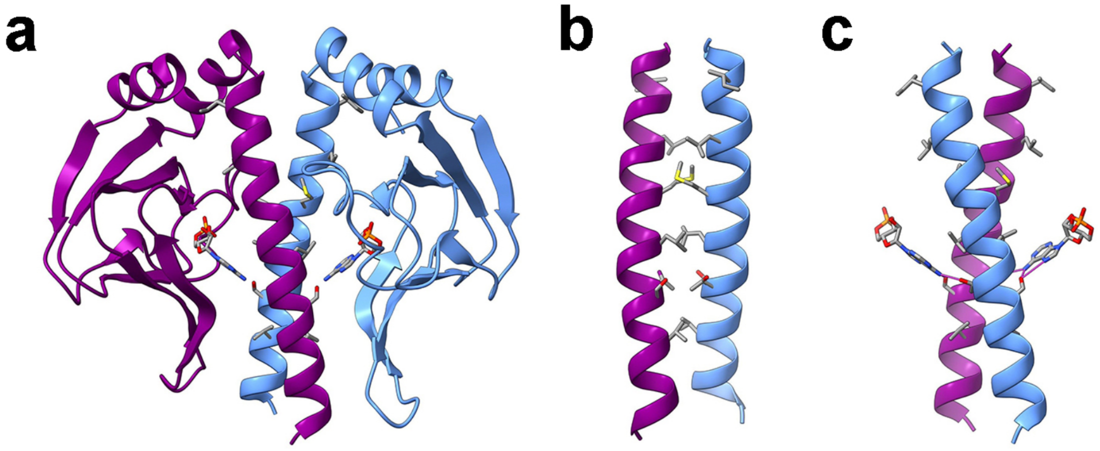


**Fig. S7** Structural models of CAP protein from E. coli. **a** Structure of a CAP dimer in the cAMP bound form (PDB: 1G6N) [2]. **b** The C-helix interface that modulates CAP dimerization. **c** Interaction of residues of the C-helices of CAP with cAMP.

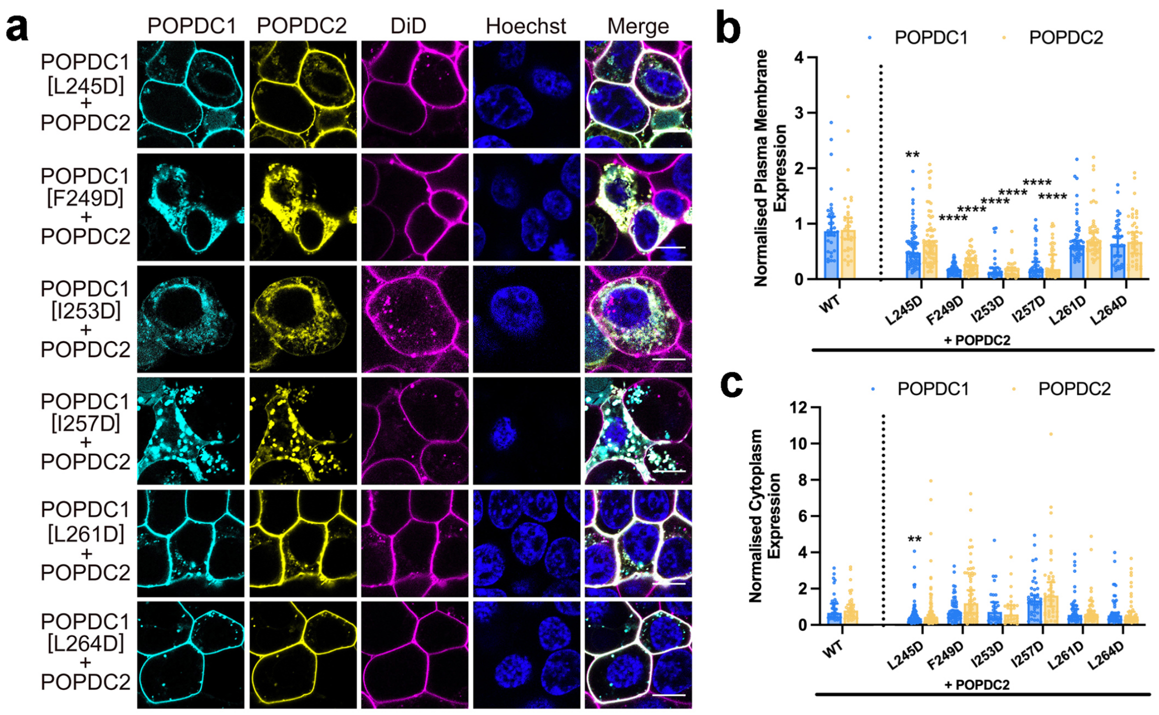


**Fig. S8** Membrane trafficking after co-transfection of αC-helix mutants of POPDC1 and wildtype POPDC2. **a** POPDC2-EYFP was expressed in HEK293 cells with wildtype POPDC1-ECFP and a series of constructs in which one of the conserved hydrophobic residues within the αC-helix had been mutated to an aspartic acid. Scale bar: 10 µm. **b** and **c** Quantification of the normalised expression levels of POPDC1-ECFP and POPDC2-EYFP in the **b** plasma membrane and **c** cytoplasm as a function of the different POPDC1 mutations. Total number of cells analysed: WT n= 34, L245D n = 71, F249D n = 56, I253D n = 27, I257D n = 40, L261D n = 47, L264D n = 42. Min two transfections per group. Bars show median ± 95% CI. Groups were compared using Kruskal-Wallis followed by Dunn's test using the wildtype pair as a comparison; ** *p* <0.01, **** *p* <0.0001.

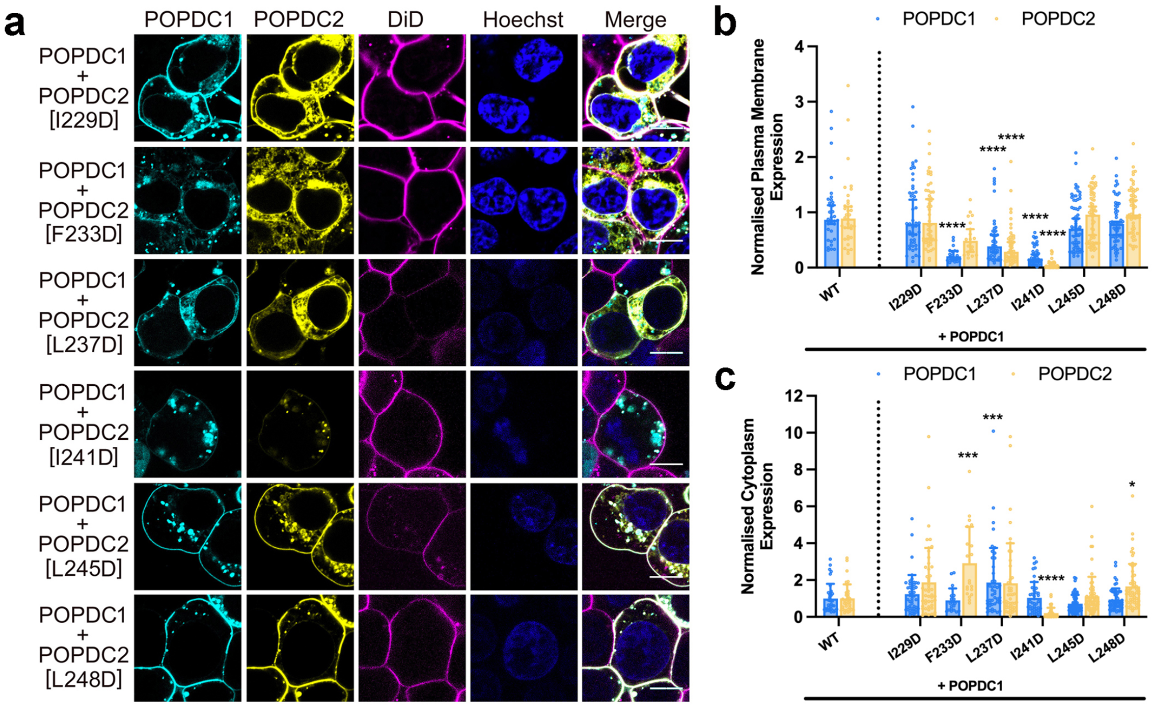


**Fig. S9.** Membrane trafficking after co-transfection of wildtype POPDC1 and αC-helix mutants of POPDC2. **a** POPDC1-ECFP was expressed in HEK293 cells together with wildtype POPDC2-EYFP or a construct in which one of the conserved hydrophobic residues within the αC-helix of POPDC2 had been mutated to an aspartic acid. The plasma membrane was marked using DiD. Scale bar: 10 μm. **b** and **c** Quantification of the normalised expression levels of POPDC1-ECFP and POPDC2-EYFP in the **b** plasma membrane and **c** cytoplasm as a function of the different POPDC2 mutations. Total number of cells analysed: WT n=34, I229D n=48, F233D n=20, L237D n=45, I241D n=42, L245D n=63, L248D n=56. Min 2 transfections per group. Bars show median ± 95% CI. Groups were compared using Kruskal-Wallis followed by Dunn's test using the wildtype pair as a comparison; * $p$ <0.05, *** $p$<0.005, **** p<0.0001.

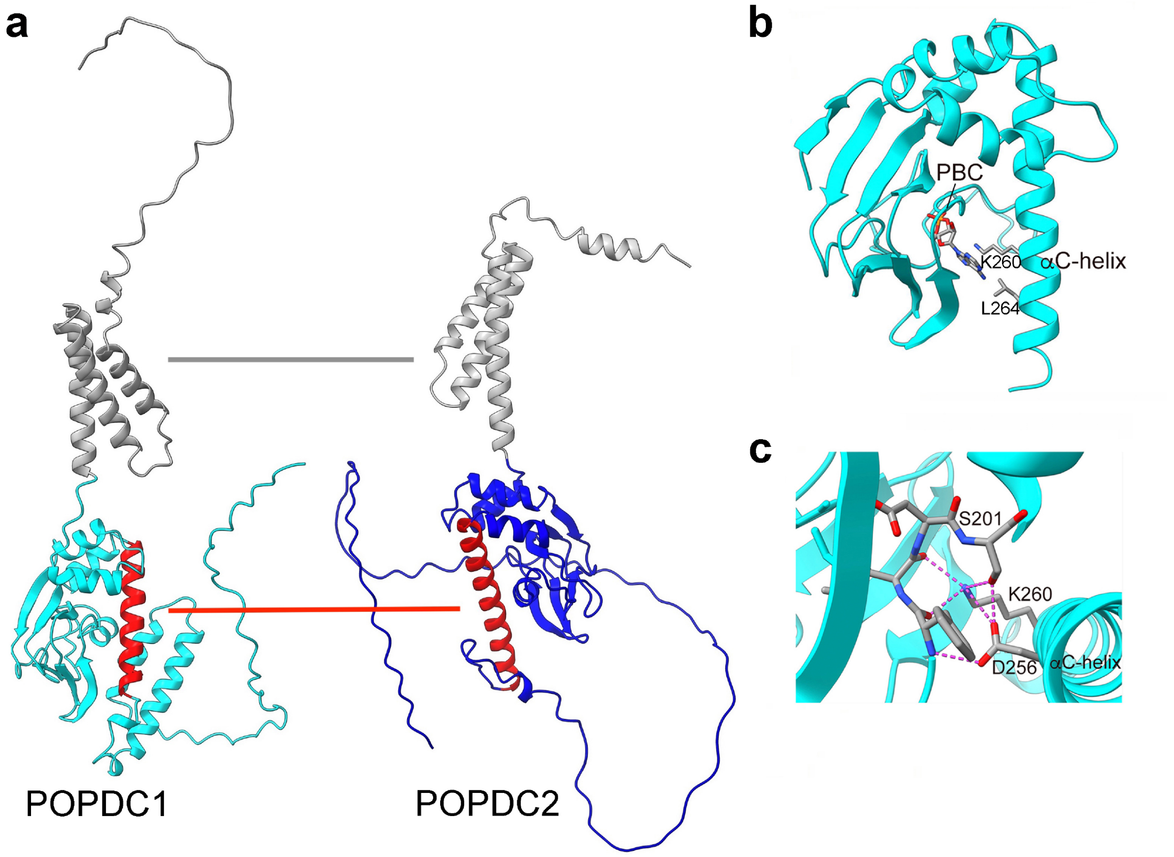


**Fig. S10** The role of the αC-helix in the function of POPDC proteins. **a** Model of the protein domains mediating the interaction of POPDC1 and POPDC2. The interaction between POPDC1 and POPDC2 is proposed to be mediated through an interface between the αC-helices (red). Another interaction via the N-terminus and/or transmembrane domains (grey) is also depicted. **b** The predicted binding mode of cAMP to the Popeye domain of POPDC1, as determined by the 3DLigandSite server [3]. The side chains of K260 and L264 are shown. **c** The side chains of the ultra-conserved S201, D256 and K260, in POPDC1 are predicted to form a H-bond network connecting the PBC of the Popeye domain with the αC-helix. Additional H-bonds formed by atoms from the peptide bond are also depicted.

**Table S1: Primer sequences used in site-directed mutagenesis of POPDC1-ECFP or POPDC2-EYFP.**

| Template | Mutation | Forward Primer | Reverse Primer |
|---|---|---|---|
| **POPDC1-ECFP** | Q153X | 5'-CGGGATCCACCGGTCGCC-3' | 5'-GATCATGCAAAACTGTCCAGTTAGTCTTCTGAAC-3' |
| | V183F | 5'-AAAAATGAAGTTCTCCTATCGAG-3' | 5'-CCCTTCAAGAGAATACTC-3' |
| | S201F | 5'-TTTATAGATTTTCCTGAATTTAGATCAACTC-3' | 5'-GGCACAGGGGTAAATGTT-3' |
| | L245D | 5’-AGAACCTTTCGACTATGAAATCTTTAGGTATCTTATTG-3’ | 5’-GATTCCAGAAAGTATGTTAATC-3’ |
| | F249D | 5’-GTATGAAATCGACAGGTATCTTATTGGAAAAG-3’ | 5’-AAGAAAGGTTCTGATTCC-3’ |
| | I253D | 5'-TAGGTATCTTGACGGAAAAGACATCACAAATAAG-3' | 5'-AAGATTTCATACAAGAAAGGTTC-3' |
| | I257D | 5'-TGGAAAAGACGACACAAATAAGCTC-3' | 5'-ATAAGATACCTAAAGATTTCATAC-3' |
| | L261D | 5'-CACAAATAAGGACTACTCATTGAATGATC-3' | 5'-ATGTCTTTTCCAATAAGATAC-3' |
| | L264D | 5'-GCTCTACTCAGACAATGATCCCAC-3' | 5'-TTATTTGTGATGTCTTTTCC-3' |
| **POPDC2-EYFP** | W188X | 5'-CGGGATCCACCGGTCGCC-3' | 5'-CTCAGGAGAGTCCATGAACTGGTATGG-3' |
| | I229D | 5’-AGAACCTTTCGACTATGAAATCTTTAGGTATCTTATTG-3’ | 5’-GATTCCAGAAAGTATGTTAATC-3’ |
| | F233D | 5’-GTATGAAATCGACAGGTATCTTATTGGAAAAG-3’ | 5’-AAGAAAGGTTCTGATTCC-3’ |
| | L237D | 5'-TAGGTATCTTGACGGAAAAGACATCACAAATAAG-3' | 5'-AAGATTTCATACAAGAAAGGTTC-3' |
| | I241D | 5'-TGGAAAAGACGACACAAATAAGCTC-3' | 5'-ATAAGATACCTAAAGATTTCATAC-3' |
| | L245D | 5'-CACAAATAAGGACTACTCATTGAATGATC-3' | 5'-ATGTCTTTTCCAATAAGATAC-3' |
| | L248D | 5'-GCTCTACTCAGACAATGATCCCAC-3' | 5'-TTATTTGTGATGTCTTTTCC-3' |

## Supplemental References

1. Felce JH, Latty SL, Knox RG, Mattick SR, Lui Y, Lee SF et al (2017) Receptor quaternary organization explains G protein-coupled receptor family structure. Cell Rep 20: 2654-2665 https://doi.org/10.1016/j.celrep.2017.08.072

2. Passner JM, Schultz SC, Steitz TA (2000) Modeling the cAMP-induced allosteric transition using the crystal structure of CAP-cAMP at 2.1 A resolution. J Mol Biol 304: 847-859 https://doi.org/10.1006/jmbi.2000.4231

3. Wass MN, Kelley LA, Sternberg MJ (2010) 3DLigandSite: predicting ligand-binding sites using similar structures. Nucleic Acids Res 38: W469-473 https://doi.org/10.1093/nar/gkq406
